# Model of P-Glycoprotein Ligand Binding and Validation with Efflux Substrate Matched Pairs

**DOI:** 10.1021/acs.jmedchem.4c00139

**Published:** 2024-03-28

**Authors:** Jay Conrad, Nick A. Paras, Roy J. Vaz

**Affiliations:** † Institute for Neurodegenerative Diseases, Weill Institute for Neurosciences, University of California, San Francisco, San Francisco, California 94158, United States; ‡ Department of Neurology, Weill Institute for Neurosciences, University of California, San Francisco, San Francisco, California 94158, United States

## Abstract

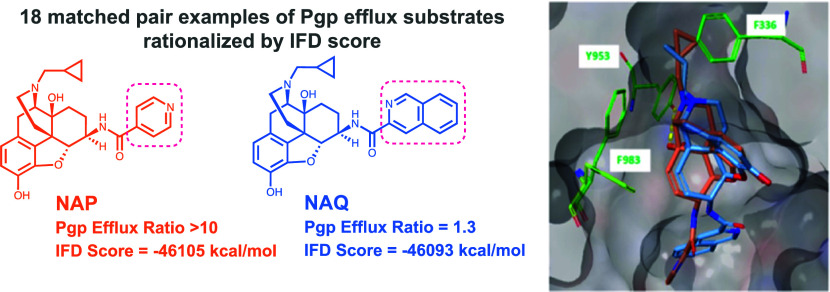

The blood–brain barrier (BBB) poses a significant obstacle in developing therapeutics for neurodegenerative diseases and central nervous system (CNS) disorders. P-glycoprotein (P-gp), a multidrug resistance protein, is a critical gatekeeper in the BBB and plays a role in cancer chemoresistance. This paper uses cryo-EM P-gp structures as starting points with an induced fit docking (IFD) model to evaluate 19 pairs of compounds with known P-gp efflux data. The study reveals significant differences in binding energy and sheds light on structural modifications’ impact on efflux properties. In the cases examined, fluorine incorporation influences the efflux by altering the molecular conformation rather than proximal heteroatom basicity. Although there are limitations in addressing covalent interactions or when binding extends into the more flexible vestibule region of the protein, the results provide valuable insights and potential strategies to overcome P-gp efflux, contributing to the advancement of drug development for both CNS disorders and cancer therapies.

## Introduction

The separation of the brain from blood by the blood–brain barrier (BBB) and the blood–cerebrospinal fluid (CSF) barrier poses a challenge to the discovery and development of therapeutics targeting the central nervous system (CNS). For most small molecules to cross the BBB, which is the major barrier for CNS penetration, they should have physicochemical properties consistent with passive membrane permeability and should not interact strongly with the efflux transporters at the BBB. Often cited as the “gatekeeper” of the BBB is the ATP-dependent drug transport protein P-glycoprotein (P-gp).^1^ P-gp is also known as multidrug resistance protein 1 (MDR1) or ATP-binding cassette subfamily B member 1 (ABCB1). P-gp transports exogenous and endogenous compounds across membranes and is expressed at many xenobiotic access points. Of particular importance to the BBB is the presence of P-gp in the capillary brain endothelium, where it acts as an efflux pump to remove small molecules from cells, thus restricting drug exposure in targeted tissues. This is a challenge for both CNS drug development and cancer therapeutic development.

The oncology drugs that are susceptible to P-gp efflux include many of the most used chemotherapy agents, such as anthracyclines, taxanes, vinca alkaloids, and many others.^2^ The discovery of the outward efflux of daunorubicin in 1973 led to the first report of chemotherapy resistance (i.e., chemoresistance).^2^ Tumors from organ tissues such as the liver, kidney, and gastrointestinal tract that normally express P-gp display an upregulation of P-gp with chemotherapy that is closely linked with increased chemoresistance.^2^ The development of P-gp inhibitors to combat efflux-mediated chemoresistance by restoring intracellular concentrations of therapeutic agents emerged as a logical approach, but thus far development of P-gp inhibitors for this purpose has not been immediately successful.^2^ The third generation of P-gp inhibitors (zosuquidar, elaquidar, laniquidar, and tariquidar) are still undergoing clinical evaluation.^2^

In this study, only molecules that behave as substrates of P-gp will be considered. The importance of P-gp efflux to drug development in both CNS and oncology indications has spurred the development of in vitro assays, quantitative structure–activity relationship (QSAR) models, and docking models. Docking models have specifically been challenged by the large, flexible, adaptive binding site of P-gp.^3^ Recently, cryo-EM structures with higher resolution have become available to elucidate the accessible conformational landscape of the P-gp binding site; these include a drug-free structure (PDB ID: 7A65),^4^ a vincristine costructure (PDB ID: 7A69),^4^ and a taxol costructure (PDB ID: 6QEX).^5^ We have utilized the taxol-P-gp costructure to validate the induced fit docking (IFD) procedure.^3−7^ In this paper, we utilized the drug-free P-gp structure as a starting point for induced fit modeling and used matched pair analysis of closely related compounds with differing P-gp efflux ratios (ERs) to determine the predictive value of the calculated model. Structure–activity relationships of P-gp efflux could then be rationalized through discrete binding events. Particular attention was given to how this computational model correlates to empirical findings relating to Fluorine incorporation or substitution, steric, or conformational switches, as these are some common strategies used by medicinal chemists to mitigate P-gp efflux.

We used compound data in which the experimental transport is attributable only to P-gp. The in vitro P-gp data for compounds in this study can be broadly described as being from two types of assays.^8,9^ The assays utilized either cells that were transfected with P-gp or cells induced to express P-gp under the influence of the substrate vinblastine.

The first set of cells consisted of Madin–Darby canine kidney (MDCK) cells or LLC-PK1 cells transfected with the MDR1 gene. The use of the ER of transport, the apparent permeability coefficient (*P*_app_)—basolateral side to the apical side divided by *P*_app_—apical side to the basolateral side, was utilized as a measure of interaction of a compound with P-gp. The efflux in colon carcinoma (Caco-2) cells was also used, at times, with and without the P-gp inhibitor GF120918 (elacridar).^8,10^

The use of the transport assay and the subsequent use of the ratio could be misleading. As shown, the passive permeability values of the compounds being compared play a significant role.^11^ Nevertheless, in this paper, we used the ERs as a measure since we compared them between compounds differing by a single chemical structure modification. Generally, a compound with a low (<5) ER was considered a weak substrate of P-gp or not a substrate at all, and a compound with a high ratio (>10) was considered a strong substrate.

The second type of cell assay utilized for the assessment of compounds is essentially only used in oncology therapy programs, as described by Hochman et al.^11^ and in more detail elsewhere.^12,13^ The assay uses the uptake difference of the compound in KB-3-1 cells and KB-V-1 cells. The KB-V-1 cells are KB-3-1 cells cultured in the presence of vinblastine, leading to >500 times higher P-gp mRNA levels. When P-gp substrates are transported out of the KB-V-1 cells, the subsequent compound effects are different in these cells when compared to those in the KB-3-1 cells. The ratio of the induction of mitotic arrest (IC_50_) in the two cell lines (IC_50_ _KB-B-1_/IC_50_ _KB-3-1_) is used instead of transport ratios.

## Experimental Computation Method

Previous modeling^3^ of compound binding showed that treating both the ligands and the protein as flexible provided promising results. The previous methodology^3^ only accounted for the flexibility of some side chains in the binding site of murine P-gp solved with inhibitors. We, therefore, decided to use the flexible receptor docking algorithm or IFD methodology,^6^ as implemented within the 2022-1 version of the Schrodinger Suite. We also utilized an extended sampling IFD protocol. Briefly, the molecules were docked flexibly using a softened energy function such that steric clashes allowed at least one ligand pose to assume a conformation close to the correct one. Side-chain rotamers were sampled, and minimization of the protein/ligand complex was performed for many different ligand poses to identify low free energy conformations of the receptor–ligand complex. A second round of ligand docking was then performed on the refined protein structures, this time using a hard potential function to further sample the ligand conformational space within the refined protein environment. Finally, a composite scoring function was applied to rank the complexes accounting for the receptor–ligand interaction energy as well as strain and solvation energies.

The cryo-EM P-gp structures were prepared using the protein preparation tools available with the Schrodinger Suite. The antigen-binding fragment (Fab) of the inhibitory antibody MRK16 or UIC2 was deleted after protein preparation from the cryo-EM structures. The extended sampling IFD score (kcal/mol) was calculated based on the energy changes in both the protein (PrimeEnergy) as well as the ligand van der Waals and Coulomb energies (GlideScore and GlideEcoul), respectively, in the following manner:^14^
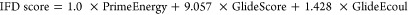
The IFD score, PrimeEnergy, and GlideScore for each ligand are reported in Tables 2–11 only for the lowest energy pose. The structures listed in Tables 2–11 were prepared within Maestro by using LigPrep. All basic N centers were protonated and used during the IFD procedure. After aligning the sequences of P-gp (PDB IDs: 7A65 and 6QEX), only the taxol structure was extracted and placed into the drug-free structure (PDB ID: 7A65). This structure, 7A65 with taxol, was utilized for all of the IFD experiments. The “transplanted” taxol was utilized to automate the definition of the IFD pocket. All other parameters for IFD were the default parameters from the “extended sampling” protocol.

**Figure 1:**
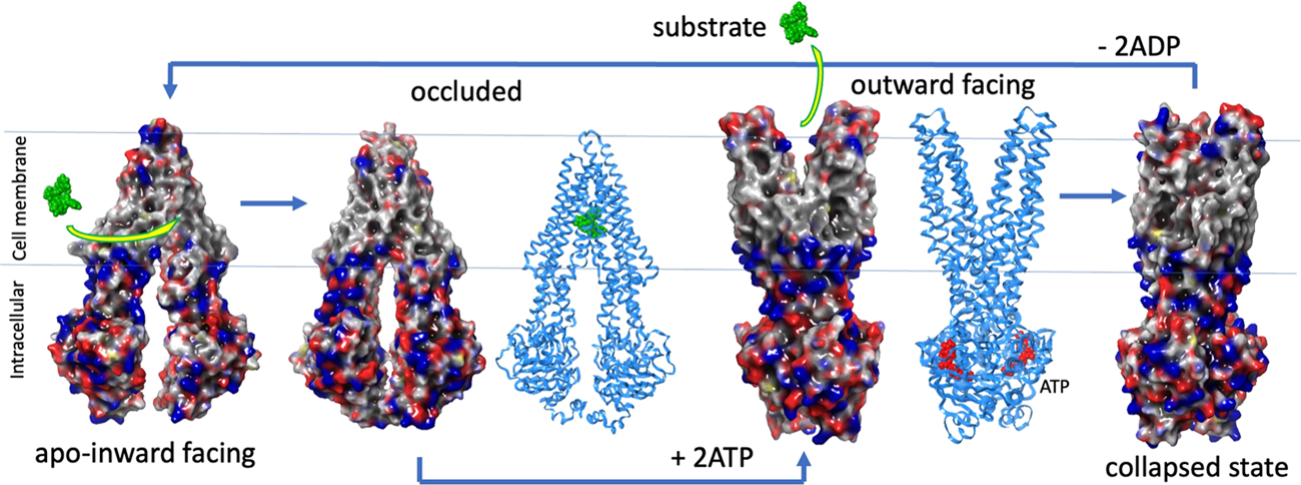
ATP-binding cassette subfamily B member 1 (ABCB1) substrate transport cycle.^5^ Upon substrate (taxol in red) binding, the inward-facing apo state changes to the occluded substrate-bound conformation. With ATP (orange), the outward-facing conformation is reached, with the substrate transported to the extracellular side. The protein collapses into the post-translocation state. Ribbon diagrams for the occluded and outward-facing conformations display the substrate bound to and ATP bound to the nucleotide-binding domains. Surfaces are colored based on residue charges.

Substrates are involved in the proposed ABCB1^5^ transport cycle (Figure 1), where P-gp undergoes major conformational changes fueled by two molecules of ATP. Upon substrate binding, the inward-facing apo state changes to the substrate-bound conformation. The change to the outward-facing conformation, whereby the substrate is released to the extracellular side, is triggered by the hydrolysis of ATP to ADP, which is bound to the two cytoplasmic nucleotide-binding domains. The outward-facing conformation then changes to the collapsed post-translocation state, which goes back to the apo conformation, thus completing the cycle. The drug-free structure (PDB ID: 7A65) resembles the substrate-bound occluded conformation rather than the apo conformation^4^ and thus was the preferred starting conformation for our substrate-docking work.

**Figure 2:**
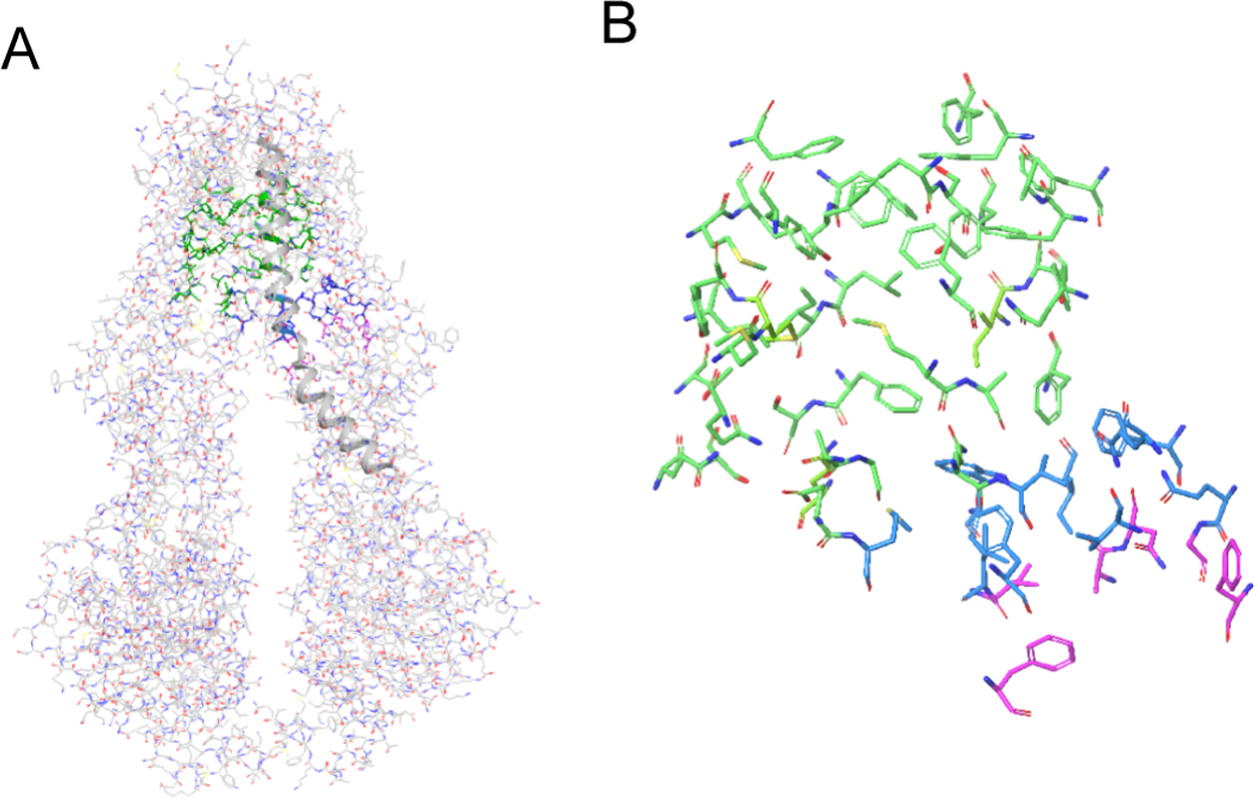
Drug-free p-glycoprotein (P-gp) cryo-EM structure (PDB ID: 7A65).^4^ (A) Binding pocket (green), the vestibule (blue), and the tunnel (cyan) were together with TM12. The antibody binding to the extracellular portion was deleted. (B) Binding pocket, the vestibule, and the tunnel were alone. The binding pocket is defined by the residues 3.5 Å away from taxol in the cryo-EM structure (PDB ID: 6QEX).^5^

Based on the analysis performed by Nosol et al.^4^ on the cryo-EM structures available, there are three subpockets in P-gp in the inward-facing occluded state (Figure 2A) – the primary binding pocket or central cavity, the vestibule, and the access tunnel (Figure 2B). The plasticity of each of the three subpockets is different in terms of accommodating a substrate in the occluded state. The subpockets play a large role in the use of the IFD method. Only four transmembrane helices (TMs) undergo major rearrangements: TMs 4, 9, 10, and 12. TMs 4 and 10 change from a kinked to a straighter conformation, whereas TMs 9 and 12 change their position and degree of bending.^4^ TM9, in particular, does not line the central cavity but rather obstructs the access tunnel and vestibule and can fully shift only when a substrate is bound in the central cavity. The access tunnel is surrounded by TMs 5, 7, 8, 9, and 12, and a molecule protruding into the vestibule or access tunnel does not allow the necessary movement of TM9 for the transport reaction to proceed.^4^ For display purposes, the residues within 3.5 Å of taxol placed in the 7A65 structure, as they occur in the three subpockets as well as the TMs to which they belong, are shown in Figure 2B and listed in Table 1. Since taxol binds only in the central cavity, most of the residues in the vestibule and access tunnel are not shown in Figure 2B or listed in Table 1.

**Table 1:** Transmembrane Helices (TMs) and the Corresponding Residues in the Three Subpockets^a^

TMs	central cavity	vestibule	access tunnel
TM1	L65, M68,		
TM3	Q195, S196,		
TM4		W232, L236	I235, F239
TM5	F303, I306,	I299	A295, N296
TM6	L339, I340, F343, S344,		
TM7	Q725, F728,		
TM8	F759	F770, Q773	G774, F777
TM9		V835, Q838	
TM10	A871, G872,	M876	
TM11	M949, Y950, Y959		
TM12	F983, M986, A987, Q990	V991, F994	

a TMs 4, 9, 10, and 12 are involved in the change in conformation from occluded to collapsed states. TM9 seems to play the largest role in this change.

The lowest energy-scored IFD poses for most molecules in Tables 2–11 fit into the primary binding pocket, except as mentioned in the analysis section of each set. When the molecules interacted with more than a single residue in either the vestibule or tunnel regions, the IFD score could be less predictive since protein backbone movement is not considered (but possible in the vestibule and access tunnel) by the IFD method. For a matched pair of molecules, a stronger interaction with the P-gp binding pocket and correspondingly a lower energy score would indicate a substrate and a higher (less negative) energy for the nonsubstrate.

To validate docking and IFD, we utilized the cryo-EM structures of the available substrates vincristine and taxol. The structure with vincristine could not be used for validation and is described in the Limitations section. We decided not to use the structures of the inhibitors since they contained multiple copies of the inhibitors—one in the primary binding pocket and others spanning the binding pocket, as well as the vestibule or access tunnel in some cases. While Nosol et al. stated that the copy in the binding pocket was better defined with better interactions, it was uncertain if the second copy influenced the conformation of the first copy or the protein itself.^4^ Also, the two copies did demonstrate interactions with each other.^4^ For example, in tariquidar (PDB ID: 7A6E)^4^ and encequidar (PDB ID: 7O9W),^15^ the central dimethoxy phenyl ring of the molecule in the binding cavity has a π-stacking interaction with the quinoline ring of the second molecule, which extends into the vestibule and the access tunnel with a distance of 3.8 Å between the centroids of these ring systems. It is uncertain what the consequences of such interactions between the inhibitor molecules and the protein could be if there is only a single copy of the molecule at lower concentrations when the molecule behaves as a substrate. Hence, the inhibitor structures were not utilized for validating the single-molecule IFD methodology.

**Figure 3:**
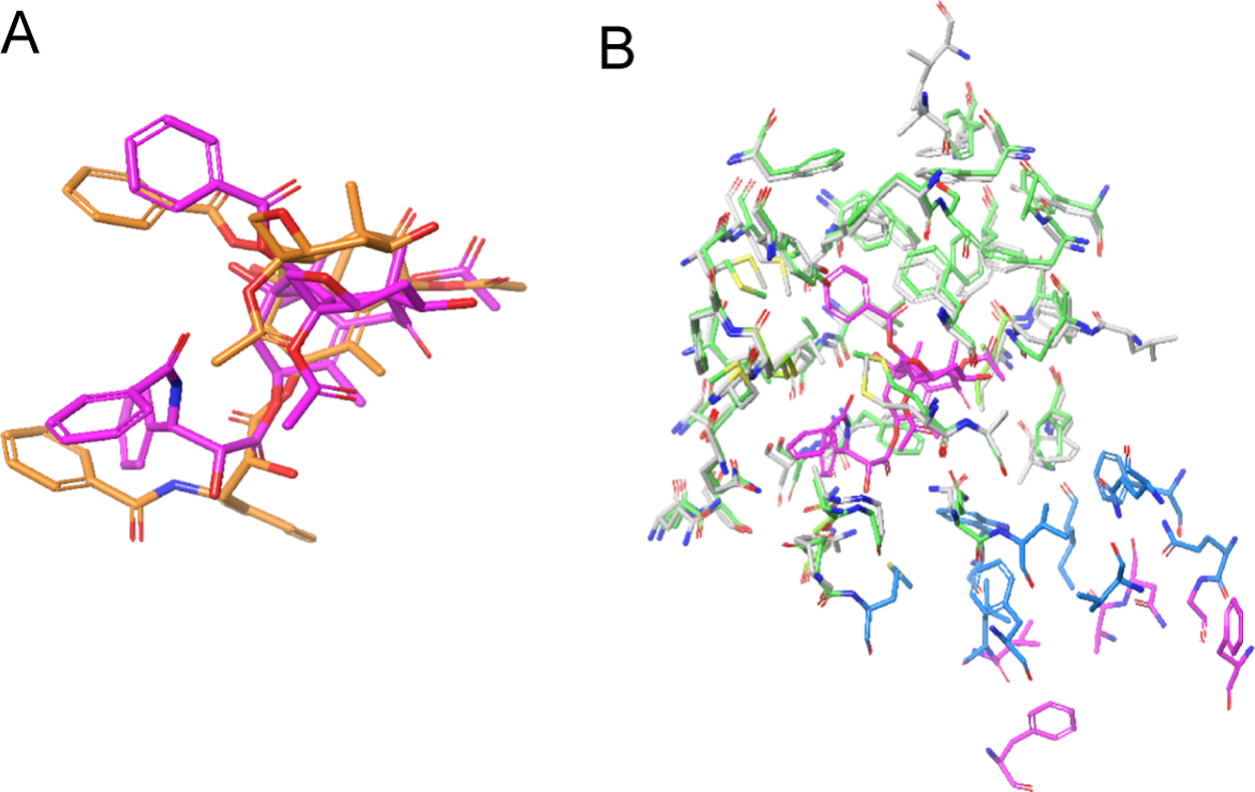
(A) Induced fit pose of taxol (orange) versus the cryo-EM structure (cyan) (PDB ID: 6QEX).^5^ The Y-tail of taxol is different in the IFD pose. (B) Residues within 3.5 Å of taxol. After the alignment of the 7A65 and 6QEX structures, taxol was moved to the vacant primary binding site in 7A65. This transplanted taxol molecule was used to define the primary binding site as well as the vestibule and tunnel regions in the drug-free pocket of the cryo-EM structure (PDB ID: 7A65).^4^

We used the IFD protocol to dock taxol into the prepared drug-free P-gp structure 7A65 to check whether the taxol 6QEX pose could be reproduced. The best overlay with the density map in the taxol-containing structure 6QEX(5) has the tetracyclic (baccatin III core) cyclooctane ring in a crown conformation. Using our modeling pipeline, the lowest IFD-scored pose reproduced the baccatin III core conformation and location in 6QEX. However, the Y-shaped tail in the fitted pose differed from the tail of taxol in the cryo-EM structure of 6QEX (Figure 3A). The density for the Y-shaped tail did not define the location of that portion of taxol in the 6QEX cryo-EM structure. The near overlay of the modeled and cryo-EM structure for the remainder of the taxol molecule gave us confidence that we could attempt to apply the method to other P-gp substrates. Additionally, Figure 3B displays the overlay of the three subpockets of the drug-free structure 7A65 with the taxol-containing cryo-EM structure 6QEX. Several residues including Y310, F343, and Q347 show side-chain movements caused by interactions with taxol. Hence, 7A65 was better than 6QEX for the purposes of the methodology described here. After aligning the backbone atoms of the 7A65 and 6QEX structures, taxol was added to the vacant primary binding site in 7A65 in exactly the same coordinates and conformation as in 6QEX. This transplanted taxol molecule was used to define the primary binding site as well as the vestibule and tunnel regions in the drug-free pocket of the cryo-EM structure (PDB ID: 7A65).^4^

## Results and Discussion

With our modeling procedure set, we then examined matched pair high and low P-gp efflux examples from the literature that came from CNS or oncology programs.^16,17^ There were several classes of molecular changes made to improve P-gp efflux: steric volume (Table 2), conformational (Table 3), modulation of H-bonding acceptors with F (Table 4), decrease of p*K*_a_ or removal of H-bond donors (HBDs) (Tables 5–7), and increase of ligand efficiency or removal of H-bond acceptors (HBAs) (Tables 8–10). Only experimental values of p*K*_a_ or *P*_app_ values, in addition to efflux-related data, are reported in the tables.

### Steric Volume Change

NAP and NAQ (Table 2) are examples of closely related compounds that, through the growth of the ligand, escape P-gp efflux. This pair of molecules was synthesized as highly selective, brain-penetrant opioid receptor antagonists with similar inhibition constant (*K*_i_) values for DAMGO antagonism in the thalamus (NAP: 4.8 nM; NAQ: 3.5 nM).^10,18^ However, while the in vitro potency of the two compounds is similar, the in vivo effectiveness for the two compounds was quite different, with NAP showing much lower efficacy. NAP also showed an ER of >10 in Caco-2 cells, confirmed by measuring apical to basolateral transport in the presence of GF120918 (elacridar), which increased permeability to that of Naltrexone (*P*_app_ <0.7 to >4 × 10^–6^ cm/s).

**Table 2:**
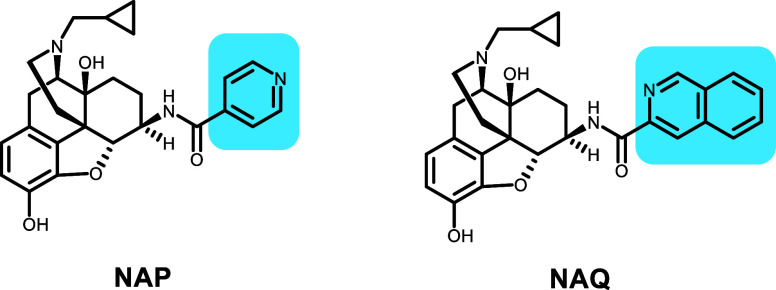
Comparison of NAP and NAQ p-Glycoprotein (P-gp) Efflux and Induced Fit Docking (IFD) Scores

compound	P-gp ER	*P*_app_ (×10^–6^ cm/s)	GlideScore	PrimeEnergy	IFD score
NAP	>10	<1	–9.46	–46 011	–46 105
NAQ	1.3	3	–9.87	–45 993	–46 098

**Figure 4:**
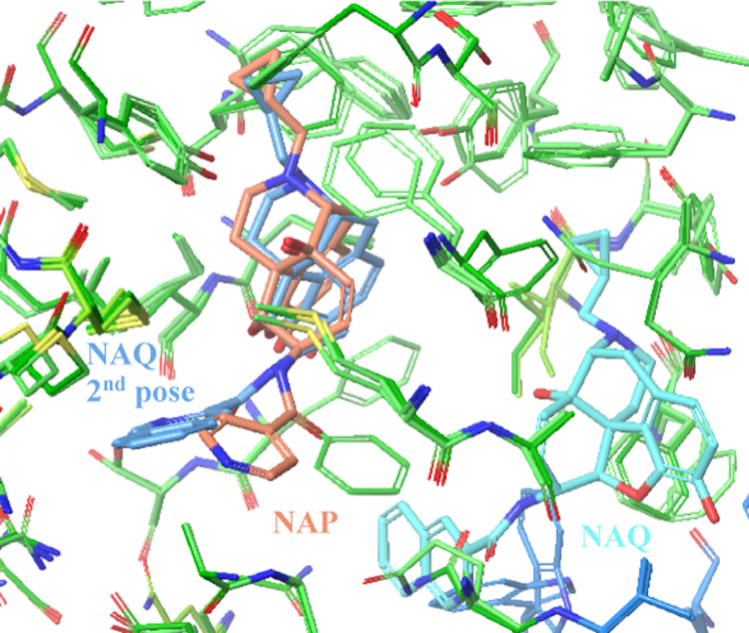
NAP (orange), with an ER of >10, has an IFD score of −46 015. NAQ (lowest-scoring pose, IFD score: −46 098, cyan; second-lowest-scoring pose, IFD score: −46 093, blue) shows no efflux. The change, a 4-pyridyl group in NAP to a 3-isoquinoline group in NAQ, cannot be accommodated and therefore displays a pose in a different location. The 3-isoquinoline group in NAQ can be accommodated in a similar location to NAP but with a different conformation and the second-lowest IFD score.

NAP has a 4-pyridine group that is replaced by a larger 3-isoquinoline group in NAQ. When IFD is performed on NAP, the lowest-scored pose (−46 105) has a 4-pyridyl group close to A871, M949, and M986. The hydroxy group displays an H-bond with Y953, and the basic N shows a cation–π interaction with F336 and F983 (Figure 4). For NAQ, the lowest-scored pose (−46 098) does not show similar interactions and binds to a different part of the primary binding site, but the second-lowest-scored pose (−46 093) shows a pose similar to that of F983 now showing an alternate conformation with no cation–π interaction. The change in the IFD score correlates well with the observed change in P-gp efflux.

### Conformational Change

Compounds **1a** and **1b** were synthesized as opioid receptor-like 1 (ORL1) antagonists.^19^ It was originally postulated that an internal H-bond^16^ would lead to an extended conformation in **1b** that would result in a different conformation and corresponding molecular shape. It was envisioned that this would correspondingly cause a decrease in the P-gp ER. While there is an internal H-bond in the lowest energy pose for **1a** and the dipoles between C=O and N–H are aligned favorably in the binding pose for **1b** (Figure S1), the interactions between **1b** in the binding pocket are more favorable than those in **1a**, as also seen in the ligand score for **1b**. However, the protein seems to be in a less stable configuration, as shown by the increased PrimeScore in Table 3, more than compensating for the increased set of interactions by the ligand. In the docked structures, there is a large difference in conformation between the molecular pair, leading to different molecular shapes and corresponding interactions with the binding pocket. As a general approach, changing the conformation of the molecule to decrease P-gp efflux might need to be balanced with potential decreases in activity toward the desired pharmacological target.

**Table 3:**
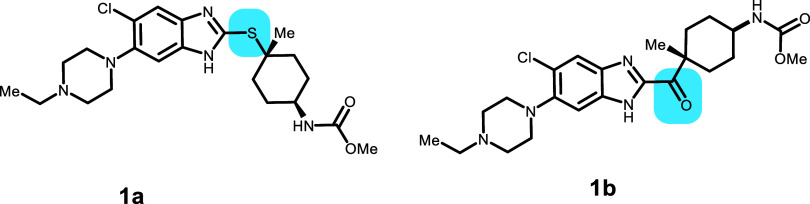
Opioid Receptor-Like 1 (ORL1) Antagonists with Conformational Changes That Restrict P-gp Efflux

compound	P-gp ER	GlideScore	PrimeEnergy	IFD score
**1a**	16	–8.92	–45 992	–46 086
**1b**	1.9	–9.68	–45 946	–46 050

### Substitutions to Decrease p*K*_a_ or Removal of an HBD

A set of six compounds, designed as kinesin spindle protein (KSP) inhibitors **2a**–**f**,^13,16^ have F incorporated to modulate the dissociation constant (p*K*_a_) of the N–H donor. The increased F substitution did not drastically affect the potency toward the desired KSP target, **2a** (2.2 nM) to difluoro **2e** (12.1 nM) until trifluoromethyl containing **2f** (110 nM). There was, however, a large impact on the MDR1 assay. In this set of data, instead of using direct efflux measurements, the effect of the compound on mitotic arrest (ratio of IC50) in KB-V-1 cells versus KB-3-1 cells was used as the measure of the compound’s interaction with P-gp, which is defined as the MDR ratio (Table 4).

**Table 4:**
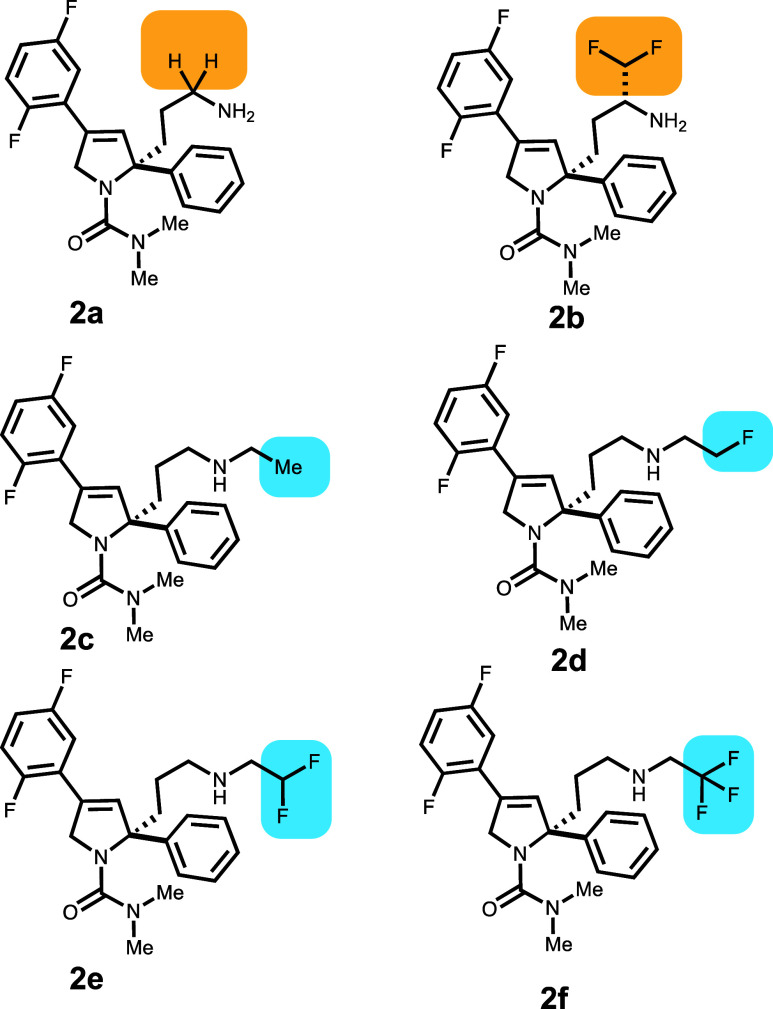
Three Kinesin Spindle Protein (KSP) Inhibitor Pairs with F Substitution Decrease p*K*_a_ and P-gp Efflux

compound	P-gp ER	MDR	p*K*_a_	GlideScore	PrimeEnergy	IFD score
**2a**	18.5	1200	10.3	–8.98	–45 884	–45 974
**2b**	2.5	5	7.0	–7.92	–45 868	–45 948
**2c**		>135	10.7	–10.18	–45 865	–45 972
**2d**		32	8.8	–9.50	–45 873	–45 968
**2e**		3	7.0	–8.59	–45 882	–45 968
**2f**		1	5.2	–9.41	–45 869	–45 964

Comparing the binding pose of **2a** with **2b**, there is no room for an (R)-CF_2_H group substituted at the carbon α to the terminal amine. The surface of adjacent residues F983 and M986 shows that the CHF_2_ group cannot be accommodated. Compound **2b**, therefore, displays a different higher energy pose (Figure S2). Even though there is an acidic residue (GLU875) in the binding cavity, basic N does not interact with it. The phenyl group is surrounded by phenyl rings from five phenylalanine residues (F72, F336, F728, F732, and F983), and the difluoro phenyl interacts with F343 and other hydrophobic residues. The higher energy binding of **2b** correlates with a large drop in MDR from 1200 for **2a** to 5 for **2b**. It is difficult to gauge how the MDR score correlates with the ER. Correspondingly, for compounds **2d** (MDR score of 32) and **2e** (MDR score of 3), which show the same IFD score, it is difficult to judge if the compounds correspond to high or low ERs.

For compounds **2c**–**2f**, fluoride is iteratively increased in the ethyl group. To better understand the poses for this series of compounds, a conformational search was done on a protonated ethyl propyl amine where the fluorination of the ethyl C2 was increased. The conformations were generated by using the MacroModel conformational search algorithm. This was followed by removing similar conformations and then optimization using M06-2X density functional theory with the 6-31G++** basis set with water as a solvent using the Poisson–Boltzmann methodology incorporated in Jaguar, part of the Schrodinger suite. The most favorable conformation for the molecules is shown in Figure S3, in which the dipoles of the C–F bond and the N–H bond are favorably aligned for the monofluorinated compound. The lowest-scoring poses of IFD molecules **2c**–**2f** were examined using this information. For compound **2c**, the distance between C–H, which would be replaced by F in **2d**, and the α-H of F983 is 2.17 Å, which is already at the van der Waals distance limits. If that H were to be replaced with F, the resulting pose would be severely sterically hindered (Figure S4); therefore, a different pose would be expected for compound **2d**. Upon examination of the pose for compound **2d**, both ethyl C2 protons are in a very tight space (Figure S5). Modifying either H to F would cause steric clashes, requiring a different pose for compound **2e**. Similarly, upon examination of the pose for compound **2e**, we found that modifying H to F would lead to a steric clash, thereby also requiring a different pose for compound **2f** (Figure S6). The trend in IFD binding energy for **2c**–**2f** correlates with a reduction in MDR from >135 for **2c** to 1 for **2f**. For compounds **2a**–**2f**, the effect of F substitution while reducing the p*K*_a_ of the basic amine, due to the larger size of F versus H, is that the docked poses also display steric effects, resulting in different interactions with the P-gp binding pocket.

Compounds **3a**–**3c** and **4a**–**4c** (Table 5) were synthesized as CNS-penetrant melanin-concentrating hormone receptor 1 (MCHR1) antagonists that were optimized to decrease P-gp efflux.^20^ The two series are different, with the N-substituted indoline ring in **4a**–**4c** being replaced with a 3-indole substituted ring system in **4a**–**4c**. Also, in **4c**, there are two simultaneous changes from **4b** compared to the other pairs in this data set. These two sets are a good example of the use of F substitution at the β position to modulate the basicity of N. The docked poses all have the compounds primarily binding in the main cavity, although there is interaction with a residue from the vestibule.

**Table 5:**
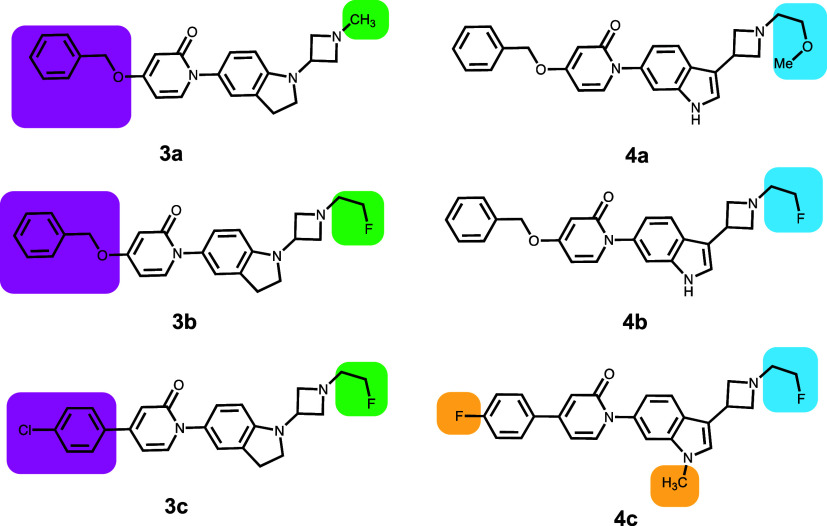
Central Nervous System (CNS)-Penetrant Melanin-Concentrating Hormone Receptor 1 (MCHR1) Antagonists

compound	P-gp ER	*P*_app_ (×10^–6^ cm/s)	GlideScore	PrimeEnergy	IFD score
**3a**	14	2.6	–10.84	–45 927	–46 032
**3b**	2.2	17.7	–10.26	–45 912	–46 016
**3c**	1	28.9	–9.53	–45 911	–46 005
**4a**	88	0.4	–9.96	–45 995	–46 088
**4b**	43	1.4	–10.35	–45 940	–46 043
**4c**	6.4	7.1	–9.94	–45 931	–46 027

In compound **3a**, the benzyl ring interacts with F314, F336, and F732. The −CH_2_F group in compounds **3b** and **3c** cannot be accommodated in the pose for **3a** due to the proximity of the Y307 ring (Figure S7). The poses for compounds **3b** (Figure S7) and **3c** show them to be displaced to accommodate the −CH_2_F group. Again, the measured P-gp efflux values correlate with an increase in binding energy. Of particular interest are compounds **3b** and **4b**—the primary differences between these two are the indoline and the indole groups. Compound **4b** shows an ER of 43, whereas compound **3b** shows an ER of 2.2, implying that **4b** should interact more strongly with P-gp. This is borne out in the stronger interaction shown by **4b** (−46 043) versus **3b** (−46 016). The indole N–H is involved in a hydrogen bond with the L339 backbone C=O in **4b** in a different pose from **3b**. Generally, while the β-F substitution affects the p*K*_a_, the F atom itself has steric effects that are manifested in the different poses in the series of compounds **2a**–**2f**, **3a**–**3c**, and **4a**–**4c**, thereby confirming that the stereoelectronic effects of F are important for modulating the P-gp efflux properties of compounds.

Azaindole compounds **5a** and **5b** were synthesized as BBB-penetrable cannabinoid 2 (CB2) agonists for pain (Table 6).^21^ Compound **5a** showed a low brain/blood ratio in rats (*K*_p_ < 0.05) and a high P-gp ER of 74. The isomeric 5-azaindole **5b** retained CB2 efficacy and had a high brain/blood ratio in rats (*K*_p_ = 1.04) and a P-gp ER of 2.9. The corresponding in vitro experiment was not described in the publication.^21^ The HBD in compound **5a** is eliminated in compound **5b**. The lowest-scoring pose for **5a** (−46 077) shows a H-bond between the azaindole NH and the side-chain amide C=O of Q946 (Figure S8); C=O is also H-bonded to NH at the 7-position of the 6-azaindole. The C=O of the amide at the 4 position is H-bonded to the amide N of the side chain of Q347. Compound **5b** in the lowest-scored pose (−46 057) lacking the Q946 interaction shifts to a different subpocket with less binding interaction (−46 057). In that pose and subpocket, there are H-bonds to both the CO as well as the NH of the side-chain amide of Q990 with the NH at the 4 position and the N at the 5-position in compound **5b**. There is also a π–π interaction between the 5-azaindole ring and F303. In this case, the p*K*_a_ increased slightly between compounds **5a** to **5b**.

**Table 6:**
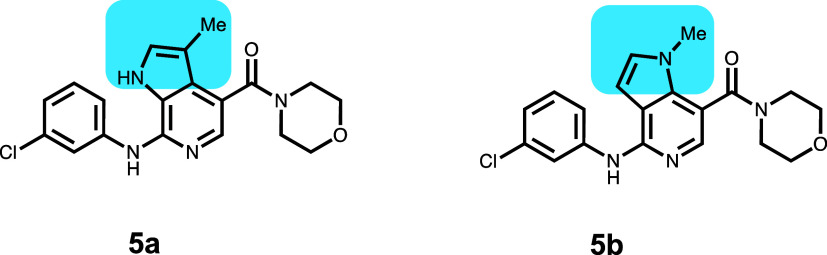
Blood–Brain Barrier (BBB)-Penetrable Cannabinoid 2 (CB2) Agonists

compound	P-gp ER	p*K*_a_	GlideScore	PrimeEnergy	IFD score
**5a**	74	5.8	–9.17	–45 980	–46 077
**5b**	2.9	6.1	–9.29	–45 965	–46 057

### Increasing Ligand Efficiency or Deletion of HBA

Compounds **6a**–**6c** come from a series of CNS-penetrant bradykinin B1 receptor antagonists for the treatment of pain (Table 7).^22^ The ER was measured in LLC-PK1 cells expressing human P-gp. The three compounds were picked for single substitution changes that have an impact on the P-gp efflux. Compound **6a** has a methyltetrazole that is replaced by a methylcarboxylate in **6b**. To check how the change affected the conformation of the molecules, two model compounds were used: 5-(3,3′-difluoro-4′-methyl-[1,1′-biphenyl]-2-yl)-1-methyl-1H-tetrazole and methyl 3,3′-difluoro-4′-methyl-[1,1′-biphenyl]-2-carboxylate. Conformations for both molecules were generated using the MacroModel conformational search algorithm, followed by the removal of similar conformations. The low energy conformations were further optimized using M06-2X density functional theory with the 6-31G++** basis set with water as a solvent using the Poisson–Boltzmann methodology. The most favorable conformation for the molecules is shown in Figure S9. Based on the calculations of the model compounds, the methyltetrazole is closer to orthogonal to the attached phenyl compared to the methylcarboxylate due to the position of the methyl groups in the two compounds. Compound **6c** has an extra methylene group and a higher ER compared to **6b**. These were then used as starting points in the IFD. Compound **6a** has the lowest IFD score (−46 064), followed by **6c** (−46 053) and **6b** (−46 039). The corresponding pose for **6a** has the center phenyl surrounded by F336 and F983. Both of the amide NHs are H-bonded to the side-chain amide carboxyl of Q725. The carboxy group of the amide adjacent to the chiral center of **6a** is H-bonded to the tyrosine hydroxy of Y310. The pose closest to this for compound **6b** is the second-lowest-scoring (also −46 039) pose. This second-lowest pose and the lowest-scored pose for **6a** are displayed in Figure S10. The difference in the torsional angle between the phenyl ring and the tetrazole defined by C(-F)–C-C=N is 113° in **6a** compared to 144° for the related torsion in the second scored pose of **6b**. Also, the *N*-methyl group on the tetrazole contributes to the larger bulk of the *N*-methyltetrazole group compared with the methyl ester in **6b**. This is the primary cause for the different poses and energy differences. Compound **6c** is displaced relative to compound **6a** to accommodate the −CH_2_CF_3_ group. Also, the distal phenyl of the biphenyl is surrounded by F983 and F336 with the NH of the amide adjacent to the chiral center, H-bonded to the side-chain amide carboxy of Q725 and the NH of the other carboxamide, and H-bonded to the tyrosine hydroxyl of Y307.

**Table 7:**
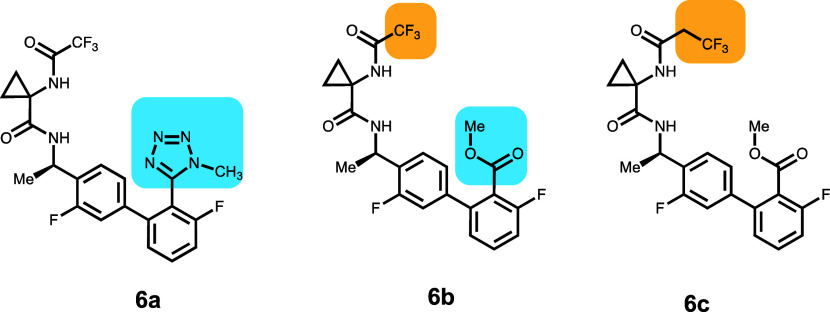
CNS-Penetrant Bradykinin B1 Receptor Antagonists for the Treatment of Pain

compound	P-gp ER	*P*_app_ (×10^–6^ cm/s)	GlideScore	PrimeEnergy	IFD score
**6a**	15.5	19	–9.36	–45 966	–46 064
**6b**	2.3	20	–10.70	–45 929	–46 039
**6c**	8.6	25	–8.93	–46 965	–46 056

The PrimeEnergy difference between **6b** versus **6a** and **6c** stems from conformation changes in protein structure that occur on binding **6b**. First, there is a π–π interaction between the indole of W232 and the aryl group bearing electron-withdrawing groups, a fluorine and methyl ester. For compound **6a**, the orthogonal orientation of the tetrazole interrupts this interaction. Second, for **6b**, there is an interaction between the trifluoromethyl amide carbonyl with the side chains of L236 and Q990. This interaction is not seen in the lowest energy pose for **6c**, as the increase in the steric size of the amide with extra methylene precludes its ability to bind in this mode. While **6b** has the most favorable GlideScore, because of the cost of adapting the protein structure to bind **6b**, the PrimeEnergy score makes this the highest energy.

Compounds **7a** and **7b** are from a series of dual serotonin and noradrenaline monoamine reuptake inhibitors (Table 8).^23^ The difference between compounds **7a** and **7b** is a tetrahydropyran to isobutyl switch and a molecular weight (MW) change of 28. The efflux values were measured in MDCK cells expressing MDR1 with an ER of 20 for **7a** and an ER of 2.7 for **7b**. The difference in the efflux may arise from the elimination of an HBA atom. However, in the lowest IFD-scored pose (−45 892), the tetrahydropyranyl O is not involved in a H-bond. The amine and carboxy of the amide are H-bonded to the amide side chain of Q990. The dichlorophenyl is also sandwiched by F303 and F343 (Figure S11). The lowest-scoring (−45 878) pose for **7b** is shifted to a less favorable subpocket where the basic amine is involved in a cation–π interaction with F343 and the dichlorophenyl surrounded by F983 and F732. The isobutyl is accommodated in a hydrophobic pocket formed by M986, F983, and F728, unlike the tetrahydropyranyl ring in **7a**, which does not immediately interact with any residues.

**Table 8:**
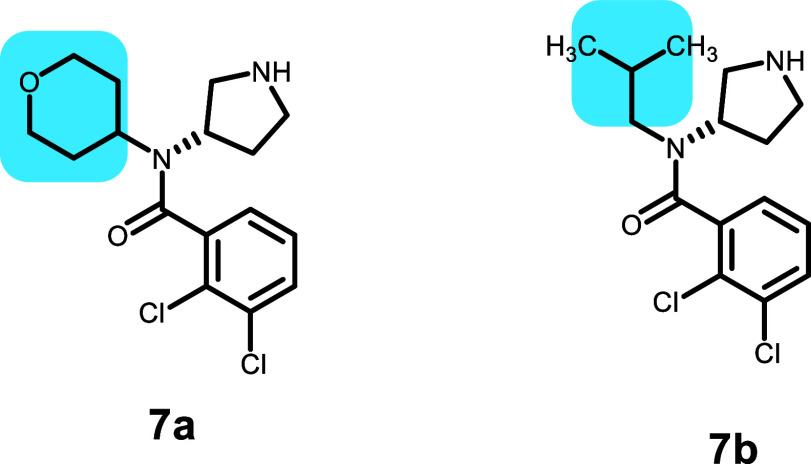
Dual Serotonin and Noradrenaline Monoamine Reuptake Inhibitors

compound	P-gp ER	GlideScore	PrimeEnergy	IFD score
**7a**	20	–7.07	–45 819	–45 891
**7b**	2.7	–7.01	–45 813	–45 878

Compounds **8a** and **8b** were synthesized as phosphodiesterase 10A (PDE10A) inhibitors for schizophrenia and were developed to be CNS-penetrant (Table 9).^24^ In this pair of molecules, increasing ligand efficiency toward PDE10A leads to a decrease in efflux. For the P-gp calculations, both the (R) and (S) isomers were used for compound **8a**, even though the chirality was reported as racemic. Both isomers of **8a**, (R)-**8a** (−46 187), and (S)-**8a** (−46 175) scored better than **8b** (−46 151). Both enantiomers of **8a** share a similar orientation, and the corresponding pose for the (S) isomer is shown in Figure S12. The quinoline ring interacts with F728 and Y307, with the quinoline N involved in a H-bond with the amide side chain of Q725. Also, the amide in the side chain of Q990 is involved in a H-bond with the methoxy oxygen attached to the quinazoline. For **8b**, the poses corresponding to the lowest IFD score, as well as the next lowest score, interact with amino acids in the vestibule (Figure S12), and the quinoline group interacts with W232, F994, F770, and F303. In addition, the amide side chain of Q990 displays a H-bond to the two O atoms attached to the quinazoline and the N atom of the quinoline. The extra hydrophobic group in **8a** adds more interaction points compared to **8b**, likely contributing to the efflux by P-gp.

**Table 9:**
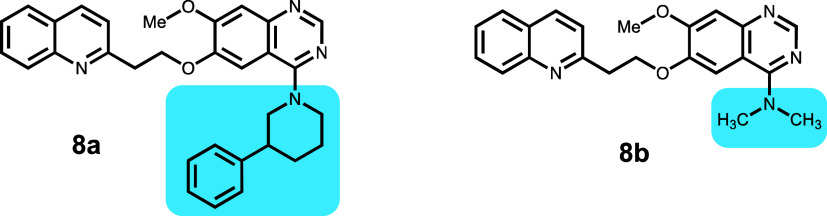
Phosphodiesterase 10A (PDE10A) Inhibitors for Schizophrenia

compound	P-gp ER	GlideScore	PrimeEnergy	IFD score
**8a**	>10	–9.61	–46 093	–46 188 (r)
**8b**	0.9	–9.38	–46 058	–46 151

Compounds **9a**–**9d** were synthesized as part of a β-secretase (BACE1) program for the reduction of β-amyloid (Table 10).^25^ From Weiss et al., we chose pairs in which single atoms or groups were modified with effects on efflux. Of the four molecules, **9a** and **9b** were considered efflux substrates since the efflux values were >10, whereas **9c** and **9d** were not considered substrates with efflux values <5. The lowest IFD scores for **9a** (−46 149) and **9b** (−46 155) are similar to each other but different from the lowest IFD scores for **9c** (−46 134) and **9d** (−46 139), which are also similar to each other. For **9a**, the pose corresponding to the lowest IFD score (Figure S13) has an H-bond from the pyridine to the Y307 hydroxy, and the adjoining C=O of the aryl amide is H-bonded to the hydroxyl group of Y310. The surface imposed by these groups (Y307 and Y310) would clash with any pyridine substitutions. Compound **9b** with an F in the 4 position changes the binding orientation of the molecule and displays a strong π–π interaction with W232 (vestibule) and a H-bond with the amide side chain of Q990. This results in a lower IFD score (−46 155) compared to **9a** (−46 146), and both compounds are efflux substrates (**9a** ER: 49; **9b** ER: 35). Interestingly, moving the F to the 2 position in **9c** greatly reduced the experimentally measured efflux (ER: 4). The IFD score for **9c** shows weaker binding (−46 134) as the interaction with W232 is less optimal. Elimination of the HBA reduces the IFD score to −46 139, and the P-gp ER was found to be 1 for **9d**.

**Table 10:**
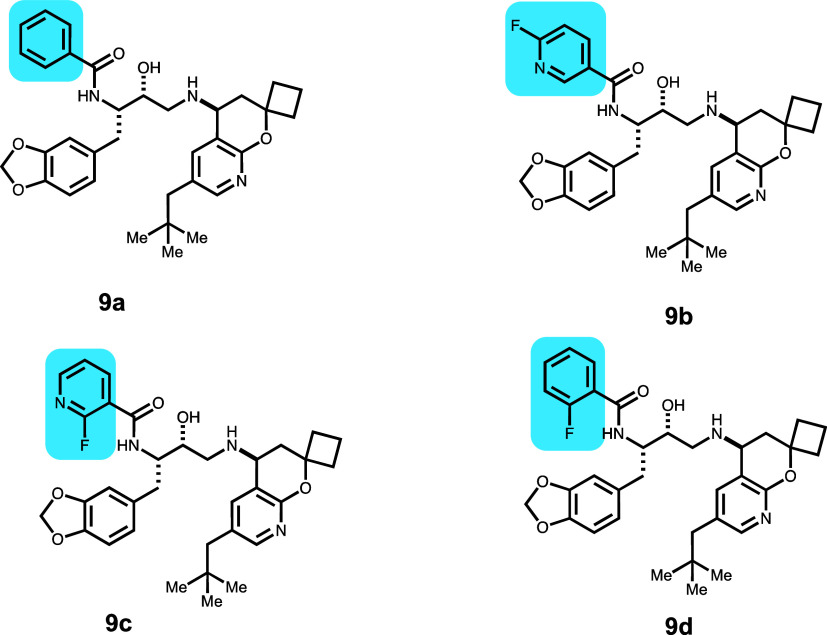
β-Secretase (BACE1) Inhibitors with Reduced P-gp Efflux and IFD Scores

compound	P-gp ER	*P*_app_ (×10^–6^ cm/s)	GlideScore	PrimeEnergy	IFD score
**9a**	49	11	–11.54	–46 030	–46 149
**9b**	35	18	–11.03	–46 044	–46 155
**9c**	4	17	–9.28	–46 045	–46 134
**9d**	1	11	–12.06	–46 021	–46 139

### Limitations

A limitation to the IFD method was found in attempts to replicate vincristine cryo-EM structure 7A69; docking of vincristine into the drug-free structure could not be replicated. Removal of vincristine from 7A69 and attempts to redock using the IFD methodology did not replicate the cryo-EM pose. The lack of agreement is largely driven by the interaction of the vincristine formyl group and Q990. Consequently, V981–F983 within TM12 (F971–P996), which line the binding pocket, are slightly displaced. This accommodates the formyl interaction and the interaction of the indole ring with the F983 side chain. Additionally, vincristine undergoes a −81° dihedral (C(=O)–C_s_–C5–C6) bond strain joining the two multiring systems as well as a shift in the lining of the pocket to accommodate this interaction. We, therefore, suggest that molecules that can form covalent interactions that induce changes in the binding pocket may not be suitable for IFD evaluation.

Compounds **10a** and **10b** were synthesized as platelet-derived growth factor β (PDGFRβ) inhibitors (Table 11).^26^ The piperidine of **10b** was modified by the addition of F β to the N and a vicinal to the ether linkage. The *trans*-R,R diastereomer **10b** has a measured p*K*_a_ of 7.4 compared to 9.3 for **10a**, and the ER of **10a** (5.7) is reduced for **10b** (1.1). The binding model of **10a** and **10b**, however, reaches into the vestibule (**10a**: Q838 H-bond; **10b**: W232 π–π), and the binding energy trend does not match the measured P-gp ER. Based on previously discussed issues, compounds that show poses docked predominantly into either the vestibule or tunnel subpockets are not as predictive.

**Table 11:**
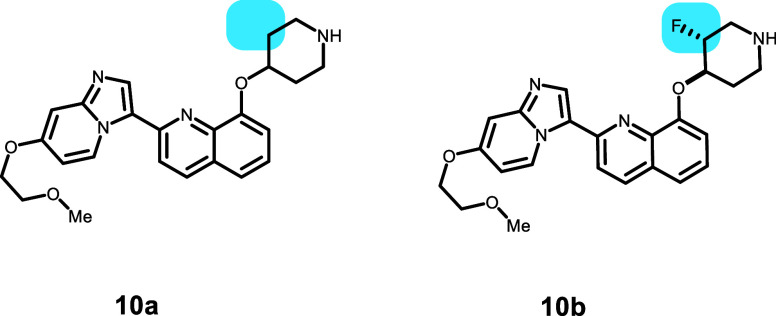
Modulation of P-gp Efflux for Platelet-Derived Growth Factor β (PDGFRβ) Inhibitors via the Incorporation of F on Piperidine

compound	P-gp ER	*P*_app_ (×10^–6^ cm/s)	p*K*_a_	GlideScore	PrimeEnergy	IFD score
**10a**	5.7	16.3	9.3	–8.76	–45 963	–46 051
**10b**	1.1	4.4	7.4	–9.99	–45 981	–46 081 (s,s)

## Conclusions

Of the 19 pairs of molecules used in this study, all 18 pairs with significant separation in efflux values were successfully differentiated using IFD into the drug-free cryo-EM structure of P-gp, 7A65. The pairs broadly fell into four classes: (1) increasing steric bulk, (2) modifying the conformation, (3) decreasing the basicity of a heteroatom or decreasing the number of HBDs, and (4) increasing ligand efficiency or deleting HBAs. For each pair examined, IFD scores were matched to the experimental efflux data.

An interesting feature that was uncovered in this study was that F substitution can alter the interaction of the compound with the protein in two ways: (1) via modulation of a neighboring group (i.e., proximal amine basicity or alcohol acidity) and its corresponding interaction or (2) changing the steric size and conformation of molecules and thus the interaction with the protein. We had assumed initially that a large portion of the examples we studied in which efflux was lowered with F incorporation would be because of attenuated hydrogen bonding interactions. However, at least in the examples we examined, it appears that the induced changes in conformation and size by substituting F for H were most influential on P-gp binding and, in some cases, completely shifted the binding to a lower energy subpocket.

**Figure 5:**
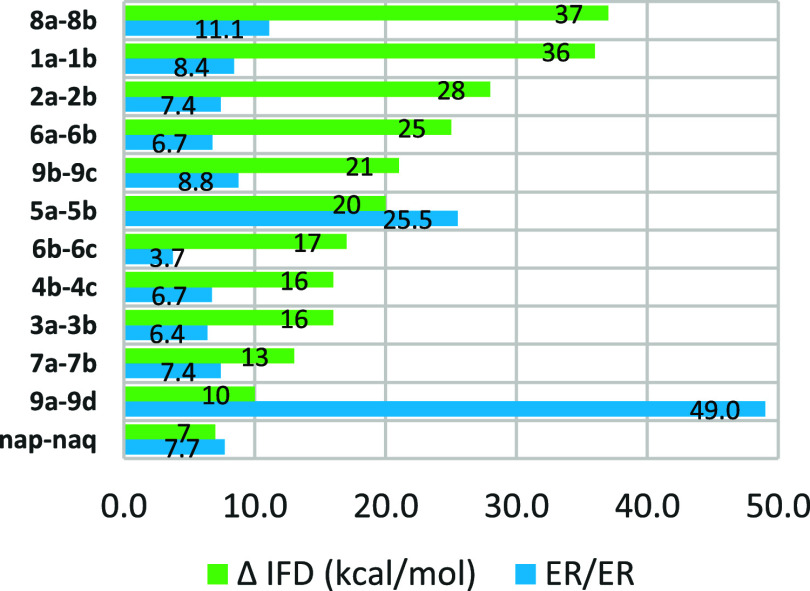
Comparison of the change in the IFD and fold change in the efflux ratio for matched pairs.

In Figure 5, we juxtapose the variations in the IFD (ΔIFD kcal/mol) with the corresponding fold changes in the ER for matched pairs. While consistent trends are observed within each chemical series, comparing IFD and ER across series is complicated by variations in assay conditions used to quantify ER. The correlation between fold change in efflux and IFD value is not linear; we ascribe this to the typical fluctuations in efflux data when categorizing compounds as substrates. Nevertheless, some overarching patterns emerge. The minimum IFD change required to elicit a discernible alteration in efflux is determined to be for the NAP-NAQ pair (ΔIFD 7 kcal/mol). Notably, even the smallest change in ER for the examined pair **6b**–**6c** (3.7 fold) is associated with a substantial IFD difference of 17 kcal/mol. On the other hand, the most significant efflux change observed in **9a**–**9d** (49 fold) corresponds to an IFD difference of 10 kcal/mol.

This method fails when covalent interactions (vincristine) are addressed or when binding bridges into the more flexible vestibule region of the protein. After developing the IFD workflow, we successfully applied this method to design molecules to evade P-gp efflux and will report these results in the future. In practice, when using this methodology, we also learned that it might take several iterations to lower the IFD score for strong efflux substrates. Examples of this use of the methodology are forthcoming.

## Supporting Information Available

Additional figures with poses and interaction maps (PDF)
SMILES molecular formula strings (CSV)
All calculated structures. (MOL)

## Supplementary Material

jm4c00139_si_002.csv

jm4c00139_si_003.mol

## Abbreviations Used

ABCB1: ATP-binding cassette subfamily B member 1
caco-2: colon carcinoma cells
cryo-EM: cryogenic-electron microscopy
ER: efflux ratio
Fab: antigen-binding fragment
IFD: induced fit docking
KB-3-1: cervical carcinoma cells
KB-V-1: cervical carcinoma cells
LLC-PKI: porcine kidney cells
MCHR1: melanin-concentrating hormone receptor 1
MDCK: Madin–Darby canine kidney cells
NAP: 17-cyclopropylmethyl-3,14β-dihydroxy-4,5r-epoxy-6b-[(4′-pyridyl)acetamido]morphinan
NAQ: 17-cyclopropylmethyl-3,14β-dihydroxy-4,5r-epoxy-6α-[(3′-isoquinolyl)acetamido]morphinan
ORL1: opioid receptor-like 1
*P*_app_: apparent permeability coefficient
PDGFRβ: platelet-derived growth factor β
p*K*_a_: dissociation constant
TMs: transmembrane helices
