# Supplementary material for: Model of P-Glycoprotein Ligand Binding and Validation with Efflux Substrate Matched Pairs
Source: J Med Chem. 2024 Mar 28;67(7):5854–65. doi: 10.1021/acs.jmedchem.4c00139 (PMC11017244; doi:10.1021/acs.jmedchem.4c00139)
Supplement: Supplementary file 1 — jm4c00139_si_001.pdf [file jm4c00139_si_001.pdf]

SUPPORTING INFORMATION

Model of P-Glycoprotein Ligand Binding and Validation with Efflux Substrate Matched Pairs

Jay Conrad*[1,2], Nick A. Paras[1,2], and Roy J. Vaz*[1,2]

[1]Institute for Neurodegenerative Diseases, Weill Institute for Neurosciences, University of California, San Francisco, California 94158, United States; [2]Department of Neurology, Weill Institute for Neurosciences, University of California, San Francisco, California 94158, United States

Roy.Vaz@ucsf.edu, Jay.conrad@ucsf.edu

Table of Contents

SUPPORTING FIGURES 2

FIGURE S1. COMPOUNDS **1A** AND **1B** 2
FIGURE S2. COMPOUND **2A** 3
FIGURE S3. MODEL COMPOUNDS TO SHOW FAVORABLE DIPOLE ALIGNMENT. 4
FIGURE S4. COMPOUND **2C** 5
FIGURE S5. COMPOUND **2D** 6
FIGURE S6. COMPOUND **2E** 7
FIGURE S7. COMPOUNDS **3A** AND **3B** 8
FIGURE S8. CB2 ANTAGONIST **5A** 9
FIGURE S9. MODEL COMPOUNDS USED TO CALCULATE THE TORSION DIFFERENCES BETWEEN A METHYLTETRAZOLE AND A METHYLESTER 10
FIGURE S10. COMPOUNDS **6A** AND **6B** ARE FROM A SERIES OF BRADYKININ B1 RECEPTOR ANTAGONISTS 11
FIGURE S11. COMPOUNDS **7A** AND **7B** ARE FROM A SERIES OF SEROTONIN AND NORADRENALINE MONOAMINE REUPTAKE INHIBITORS 12
FIGURE S12. (A) COMPOUNDS **8A** (ORANGE) AND **8B** (CYAN) WERE SYNTHESIZED AS PHOSPHODIESTERASE 10A (PDE10A) INHIBITORS 13
FIGURE S13. COMPOUNDS **9A** AND **9B** 14
FIGURE S14. LEGEND FOR 2D LIGAND INTERACTION DIAGRAMS. 15
REFERENCES 15

Supporting Figures

A

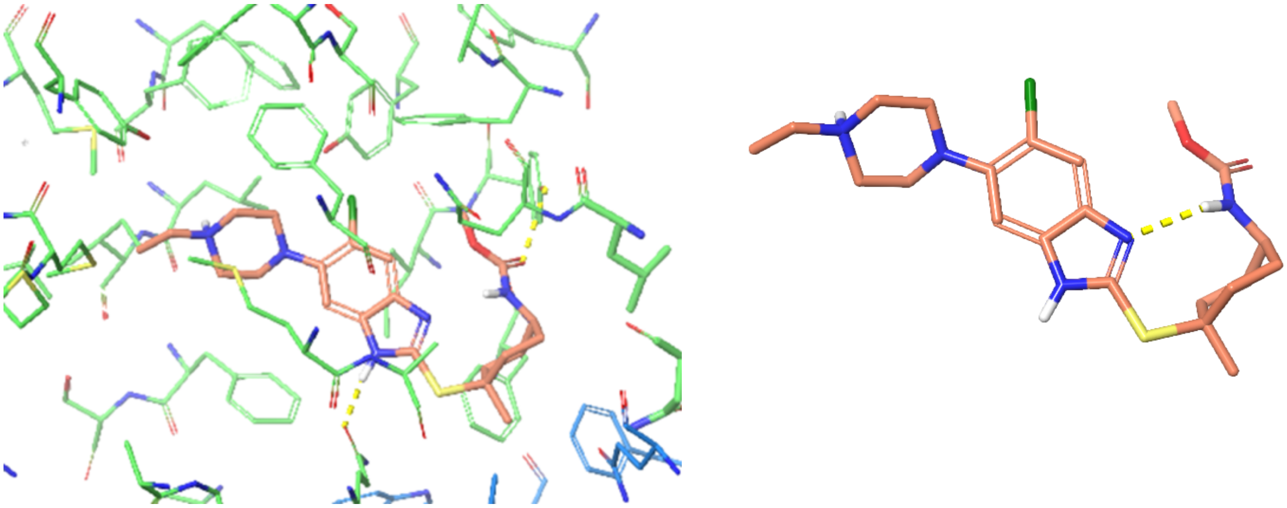

B

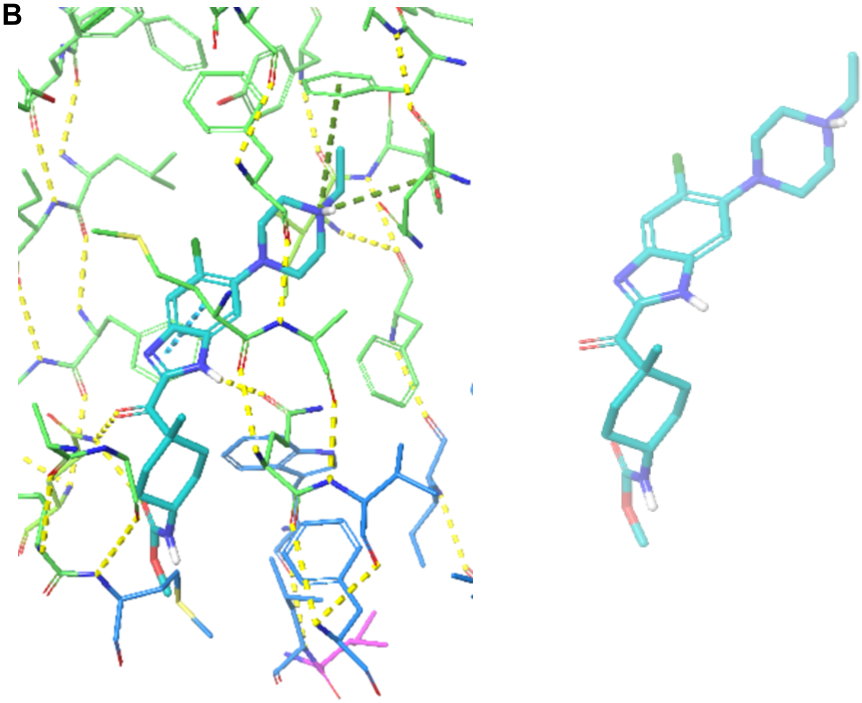

Figure S1. Compounds **1a** and **1b**: (A) Compound **1a** (orange) displays an internal H-bond but not in the manner that was postulated.[1] (B) The change from –S to –C=O does eliminate the H-bond and compound **1b** (cyan) displays an elongated conformation.

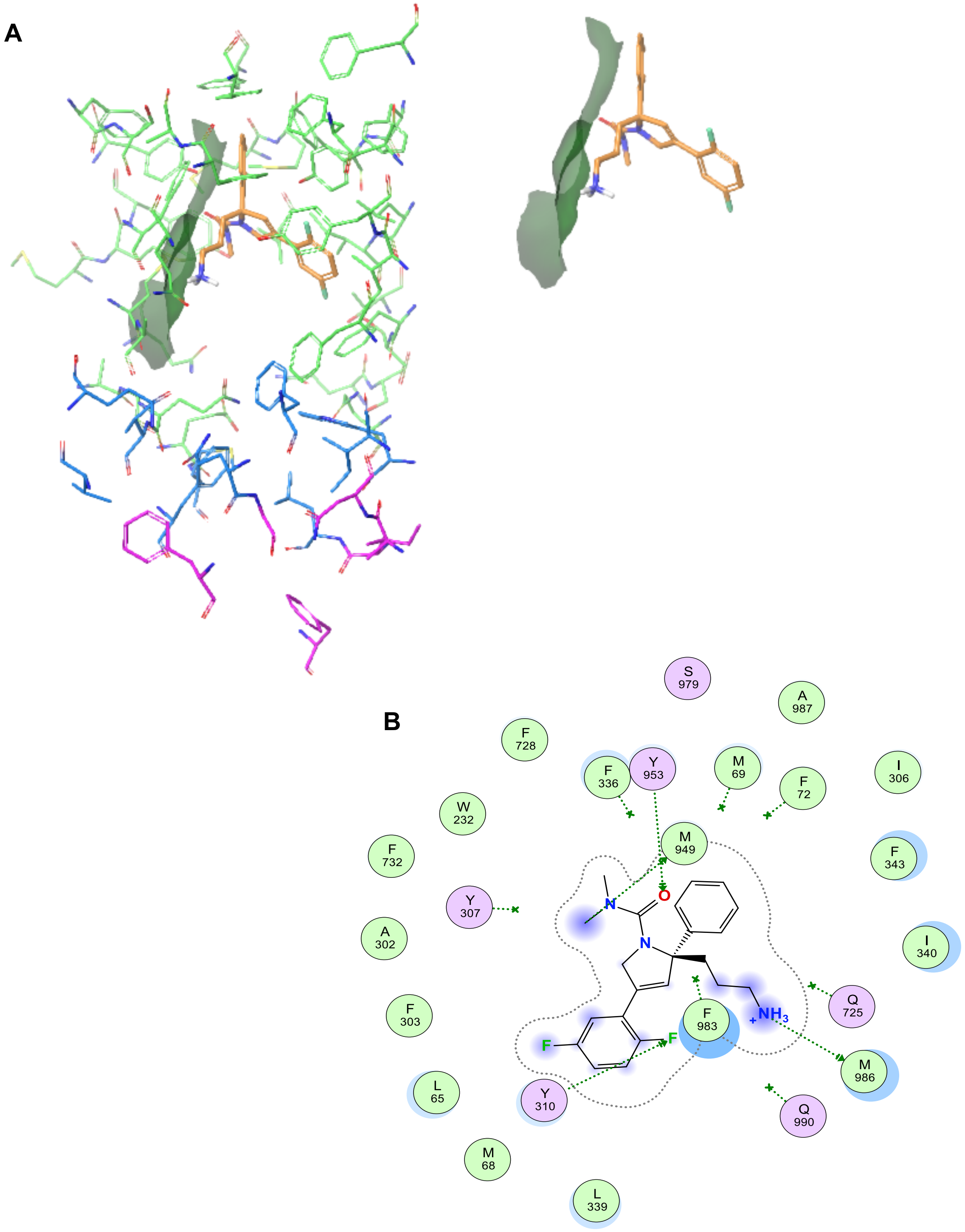

Figure S2. Compound **2a**: (A) The pose for compound **2a** displays no room for the -CF2H group in compound **2b**. The surface of the adjacent residues F983 and M986 shows that the -CF2H substitution could not be accommodated by compound **2b** in the same conformation or pose. (B) The 2D ligand-interaction diagram of compound **2a**.

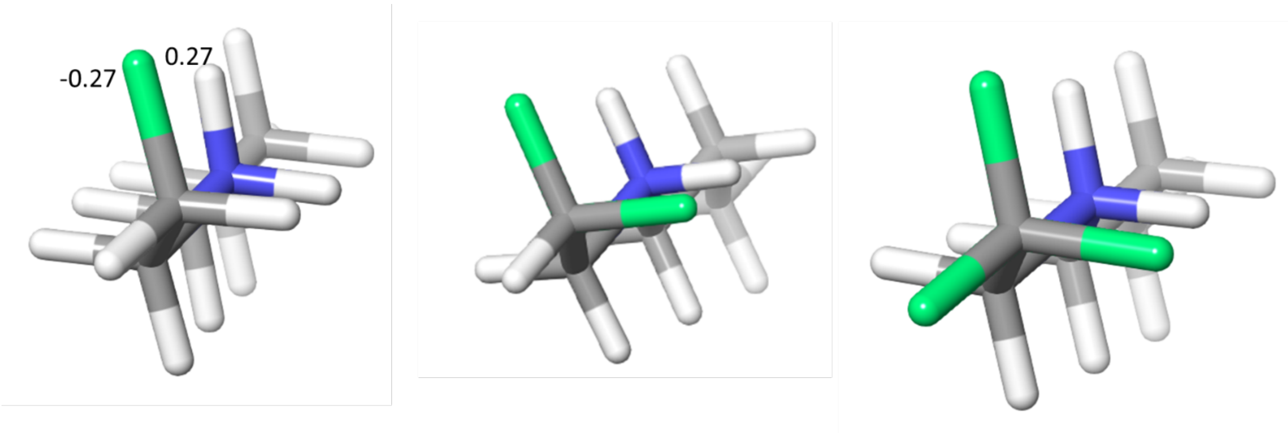

Figure S3. Model compounds to show favorable dipole alignment. The low energy conformations for mono-, di-, and tri-fluorinated ethyl propyl amine using DFT-based conformational search with solvation shows the C-F and N-H bond dipoles aligned. The conformation of the F-substituted ethyl groups helps explain the poses for compounds **2d**, **2e**, and **2f**.

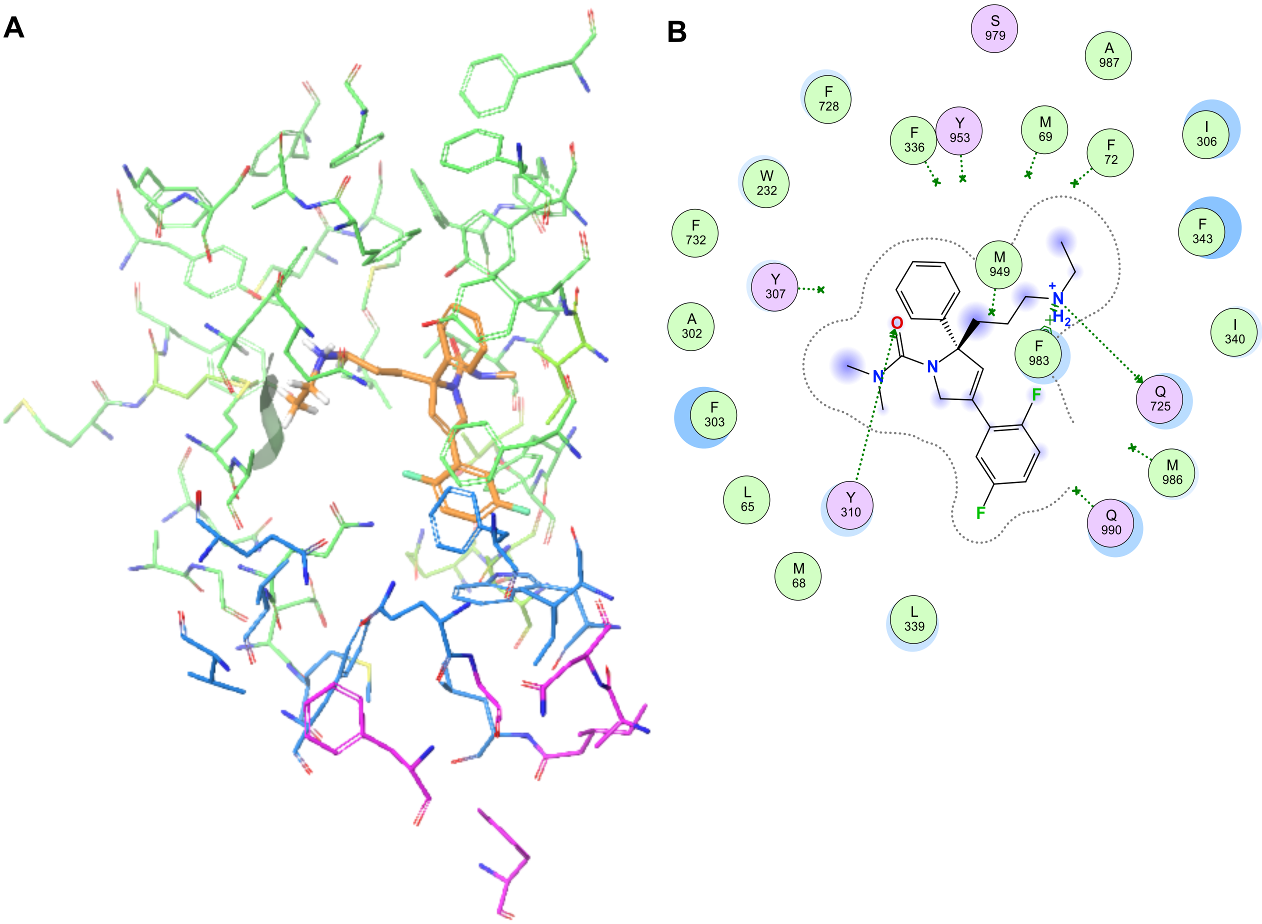

Figure S4. Compound **2c**: (A) F substitution in compound **2d** would not be accommodated in this pose due to the positions of the adjoining residues M986 and F983. The orientation of the pocket is rotated approximately 90° around the vertical axis compared to previous orientations. (B) The 2D ligand-interaction diagram for compound **2c**.

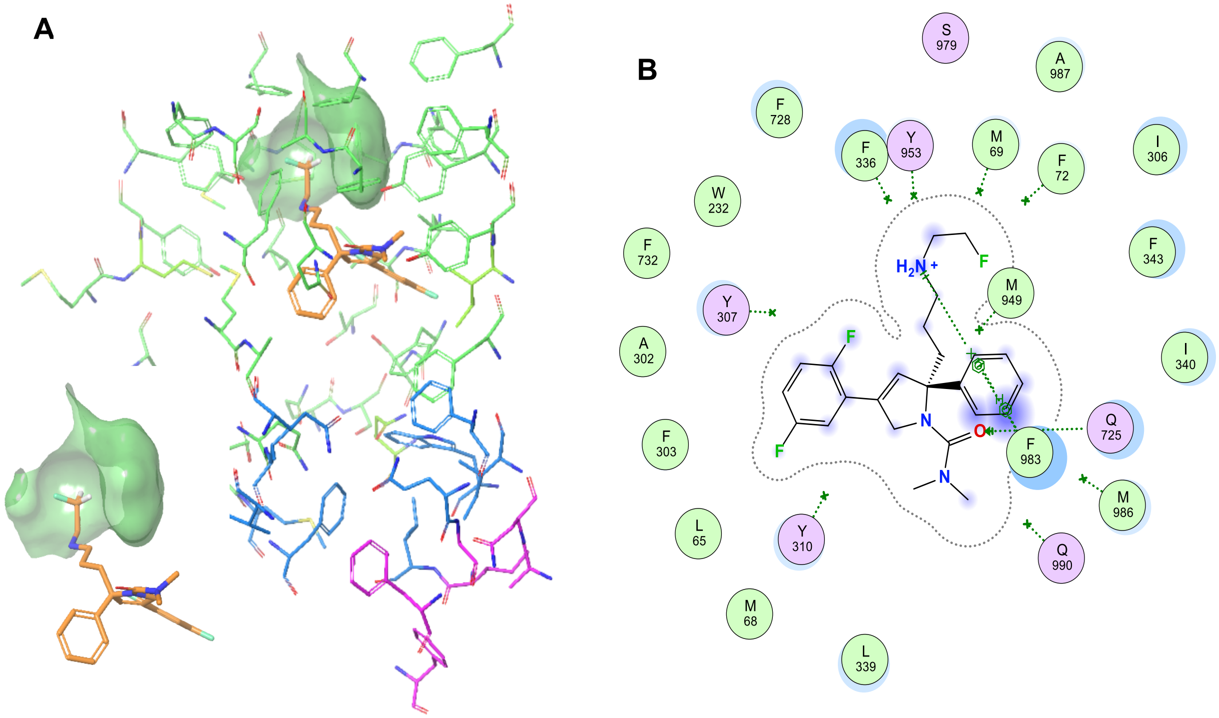

Figure S5. Compound **2d**: (A) The monofluorinated compound **2d** shows the –CH2F group in a low energy conformation but surrounded by F72, F336, and M69. The -CF2H group in compound **2e** would not be accommodated a similar pose. (B) The 2D ligand-interaction diagram for **2d**.

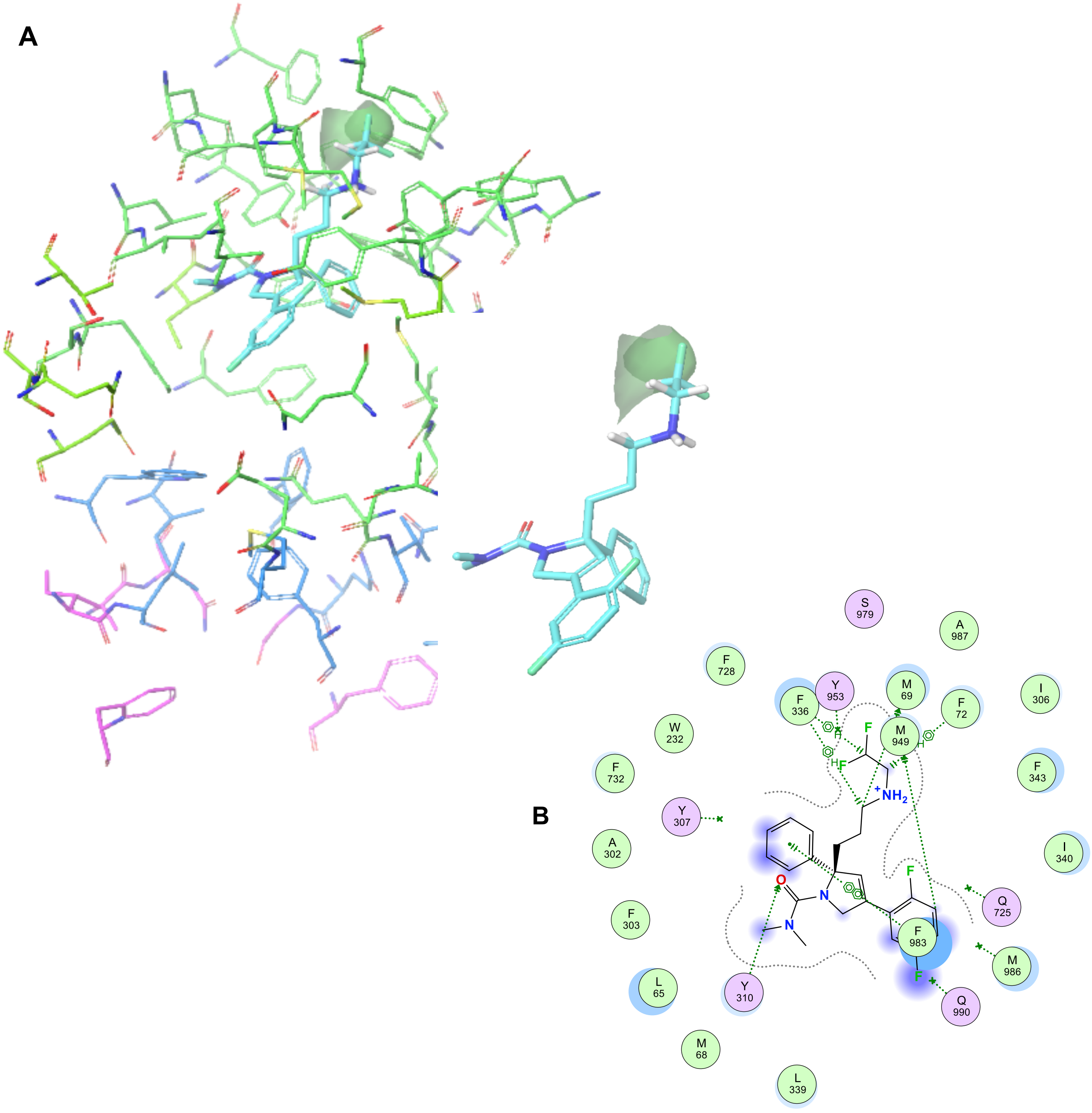

Figure S6. Compound **2e**: (A) The difluorinated compound **2e** (cyan), shows the –CF2H group in a low energy conformation. The -CF3 group in compound **2f** could not be accommodated in this pose due to the proximity of F336. (B) The 2D ligand-interaction diagram of **2e**.

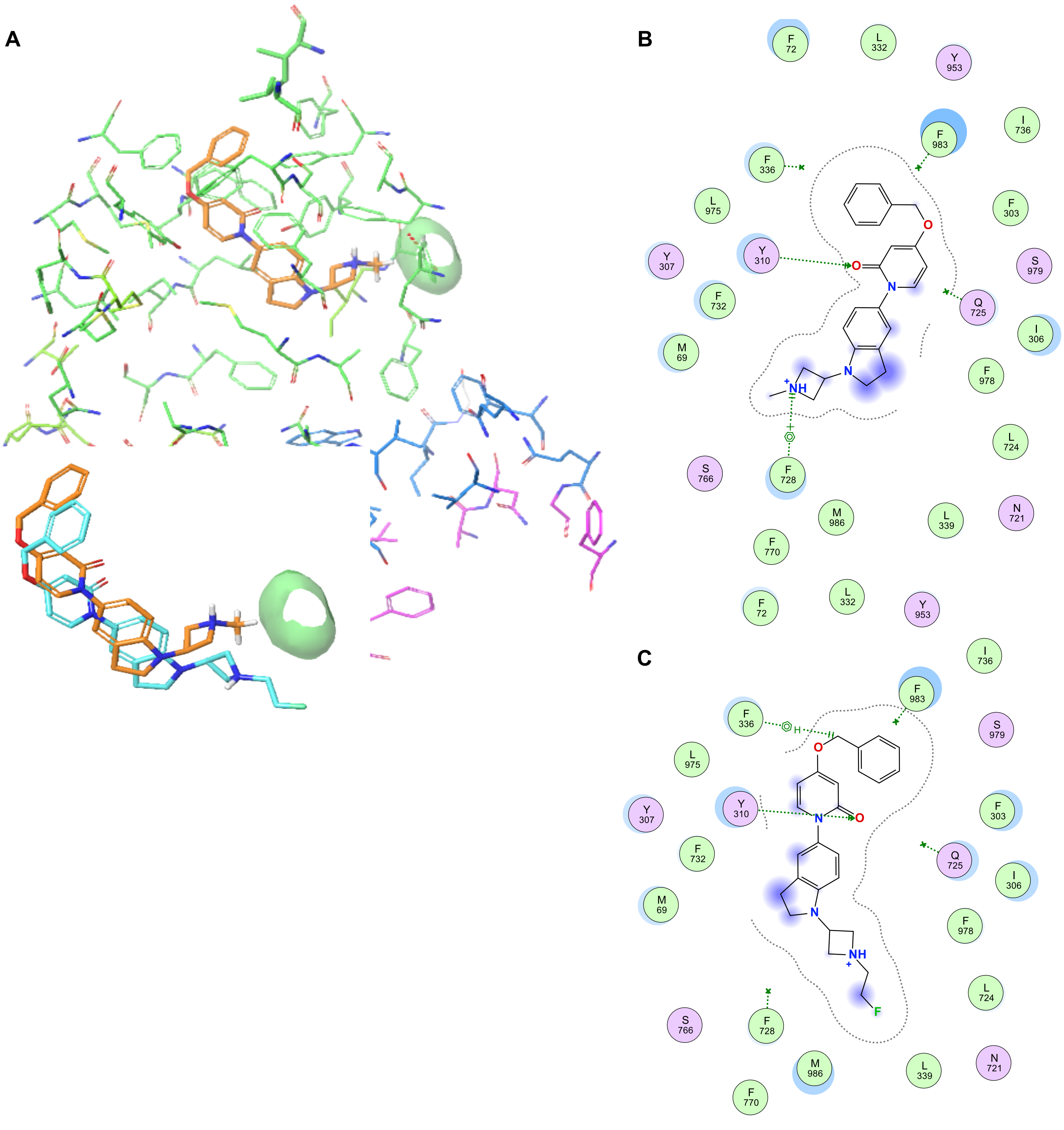

Figure S7. Compounds **3a** and **3b**: (A) The best scored pose for compound **3a** (orange) shows the -NCH3 group adjacent to the Y307 side chain (surface) and a cation-pi interaction with F728. The best scored pose for compound **3b** (cyan) adopts a similar pose, but the azetidine ring in compound **3b** displays a conformation that accommodates the steric "bump" and the molecule is slightly shifted compared to **3a**. (B) The 2D ligand interaction diagram for compound **3a**. (C) The 2D ligand-interaction diagram for compound **3b**.

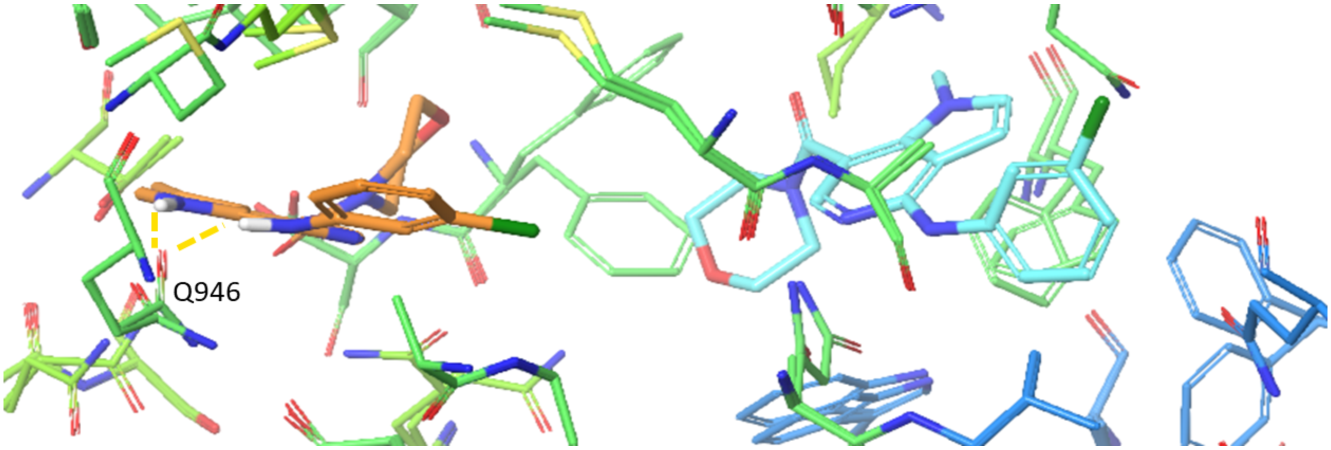

Figure S8. CB2 antagonist **5a** (orange) has an extra H-bond donor involved in a H-bond together with the exocyclic NH to the sidechain C=O of Q946, leading to a better scoring pose (-46077). The isomeric compound **5b** (cyan) binds to a different subpocket with a worse scoring (-46057) pose.

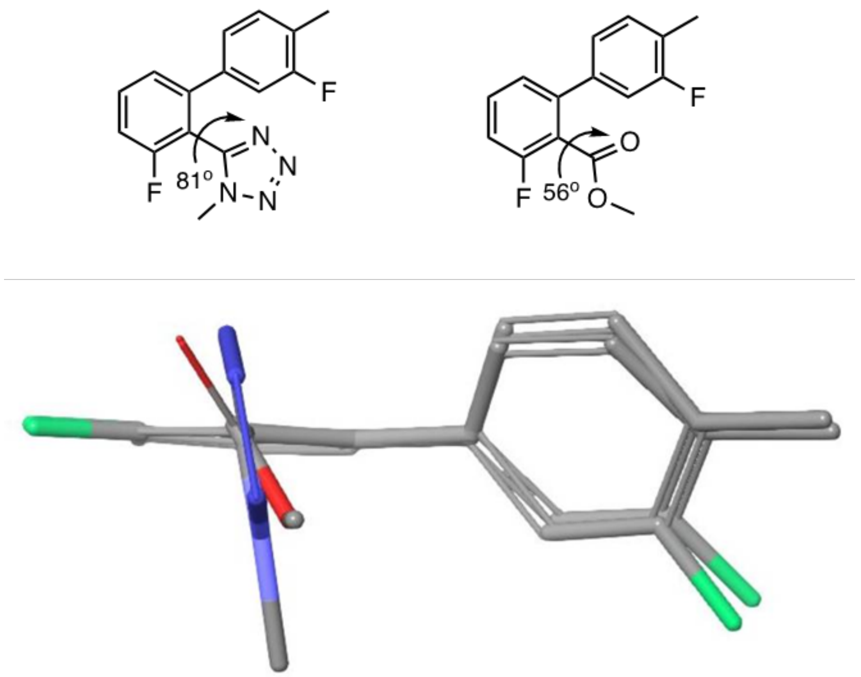

Figure S9. Model compounds used to calculate the torsion differences between a methyltetrazole and a methylester. The difference supports the observed pose difference between compounds **6a** and **6b**.

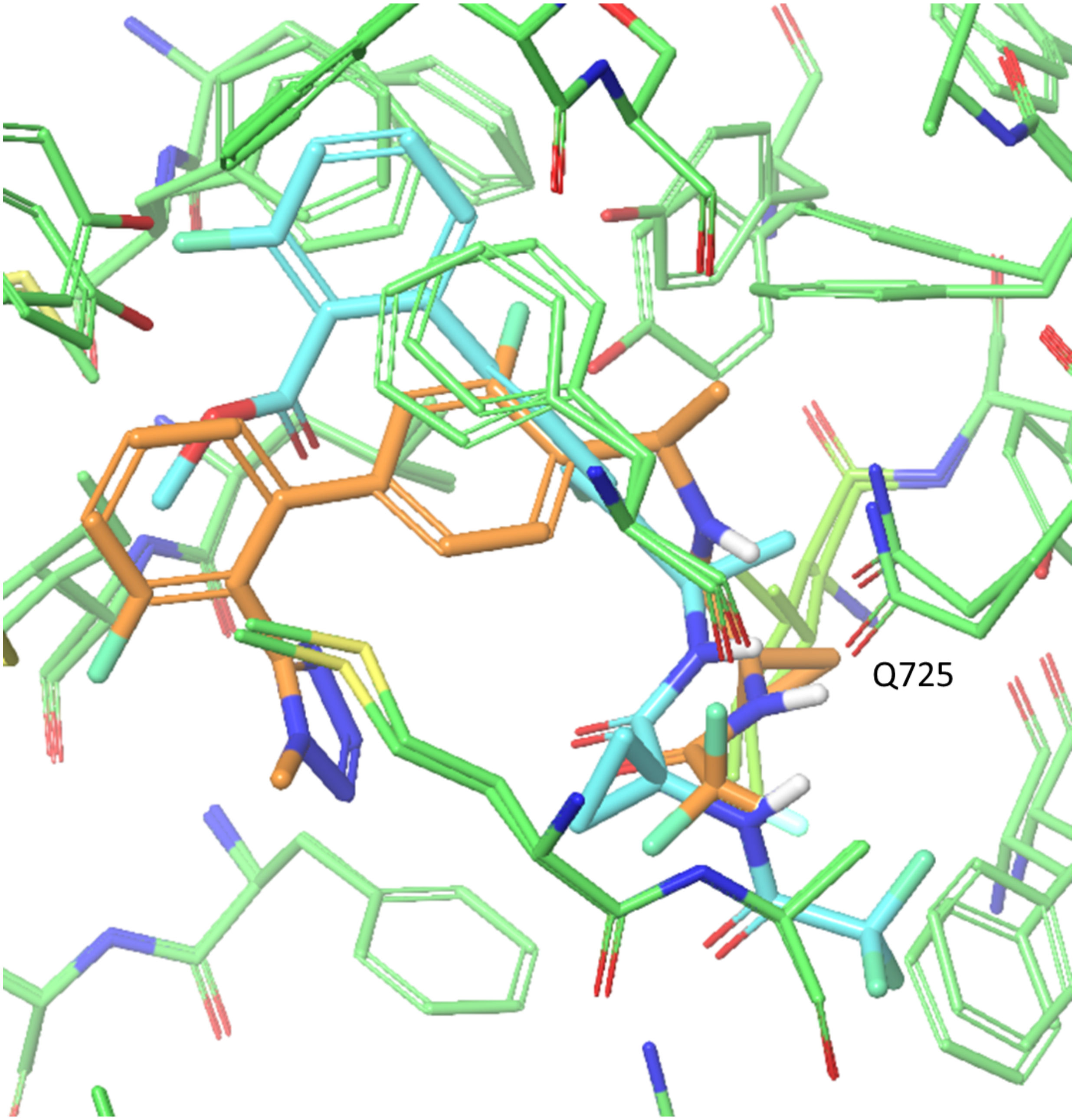

Figure S10. Compounds **6a** and **6b** are from a series of bradykinin B1 receptor antagonists. The lowest energy pose for 6a (orange, -46064) and the second-lowest energy pose for **6b** (cyan, -46039) display a similar binding mode. The pose for **6a** is stabilized by both NH groups H-bonded to the side chain amide C=O of Q725 as well as by pi-pi stacking between the biphenyl groups and the surrounding phenylalanine rings. The difference in the torsional angle between the 3-F-phenyl and the tetrazole of -67° versus -37°, respectively, for the related torsion in 6b as well as the difference in steric bulk between the methyltetrazole and the methylcarboxylate largely account for the differences in the scores and the poses.

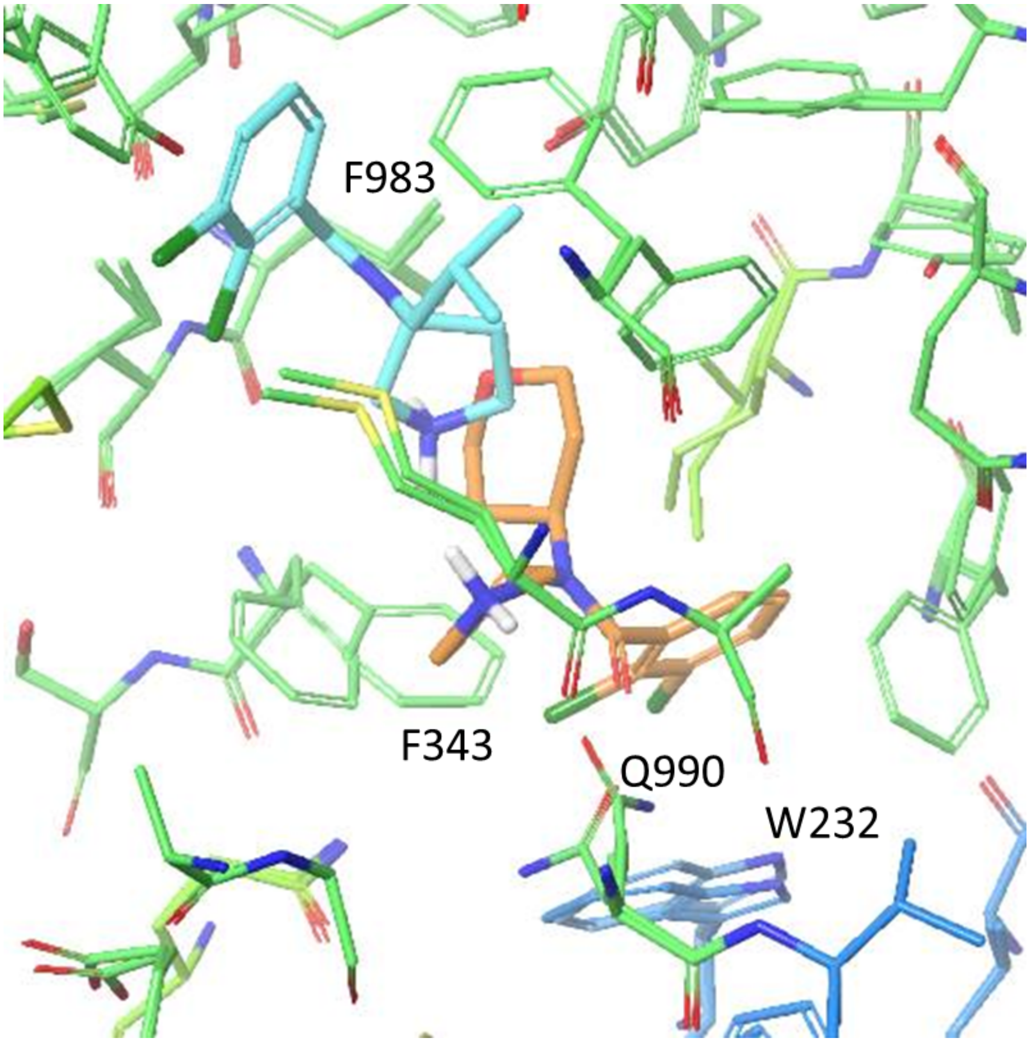

Figure S11. Compounds **7a** and **7b** are from a series of serotonin and noradrenaline monoamine reuptake inhibitors. Compound **7a** (orange) with a 4-tetrahyropyranyl group versus compound **7b** (cyan) with an isobutyl group show different lowest energy poses. Compound **7a** displays pi-stacking between the dichlorophenyl group and W232 and H-bonds with Q990. Compound **7b**, with a more hydrophobic isobutyl, displays a cation-pi interaction with F343 and a pi-pi interaction between the dichlorophenyl group and F983.

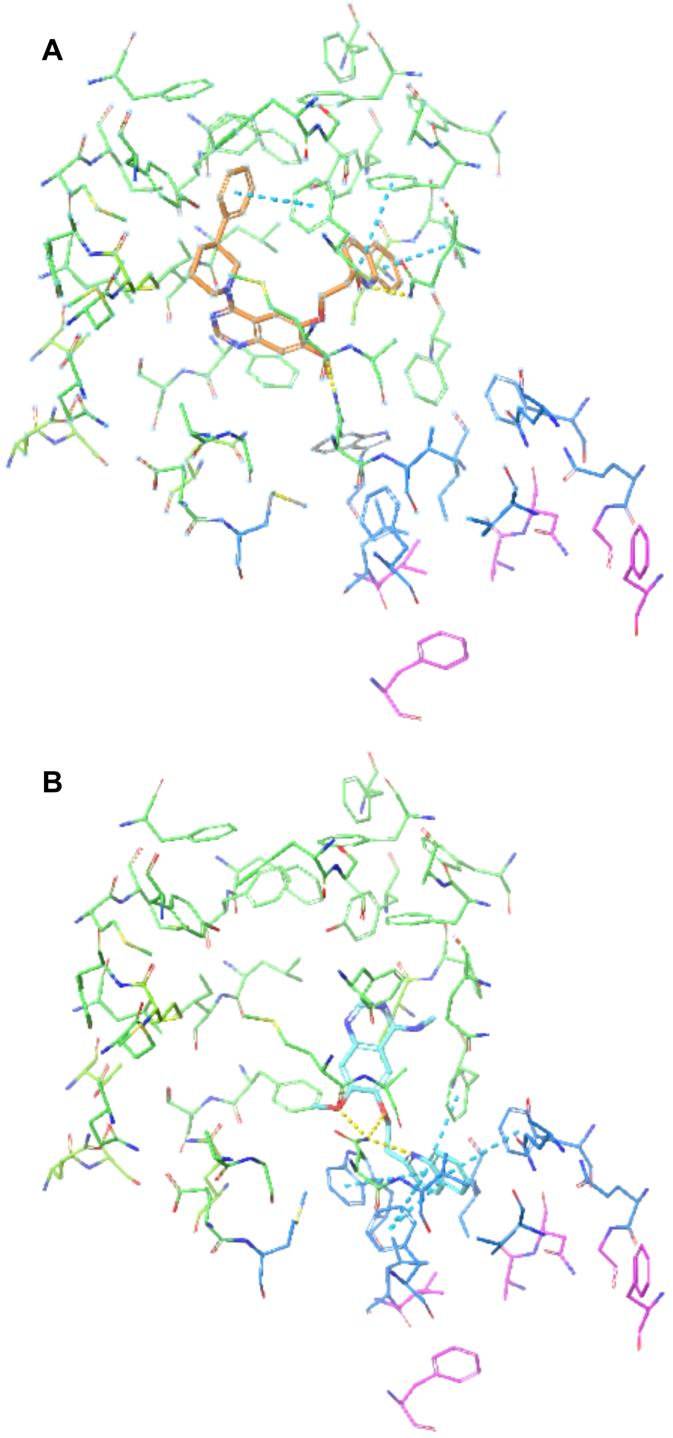

Figure S12. (A) Compounds **8a** (orange) and **8b** (cyan) were synthesized as phosphodiesterase 10A (PDE10A) inhibitors. Chirality for compound **8a** was not specified, and both enantiomers were used in the procedure. The R-enantiomer is displayed. The S-enantiomer shows a similar pose. (B) **8b** lacks the phenyl-piperazine moiety and hence has a lower MW. Pi-pi interactions contribute heavily to the lowest energy pose for **8a** (S). The quinoline ring in **8b** interacts with multiple residues in the vestibule region, mainly via pi-pi interactions.

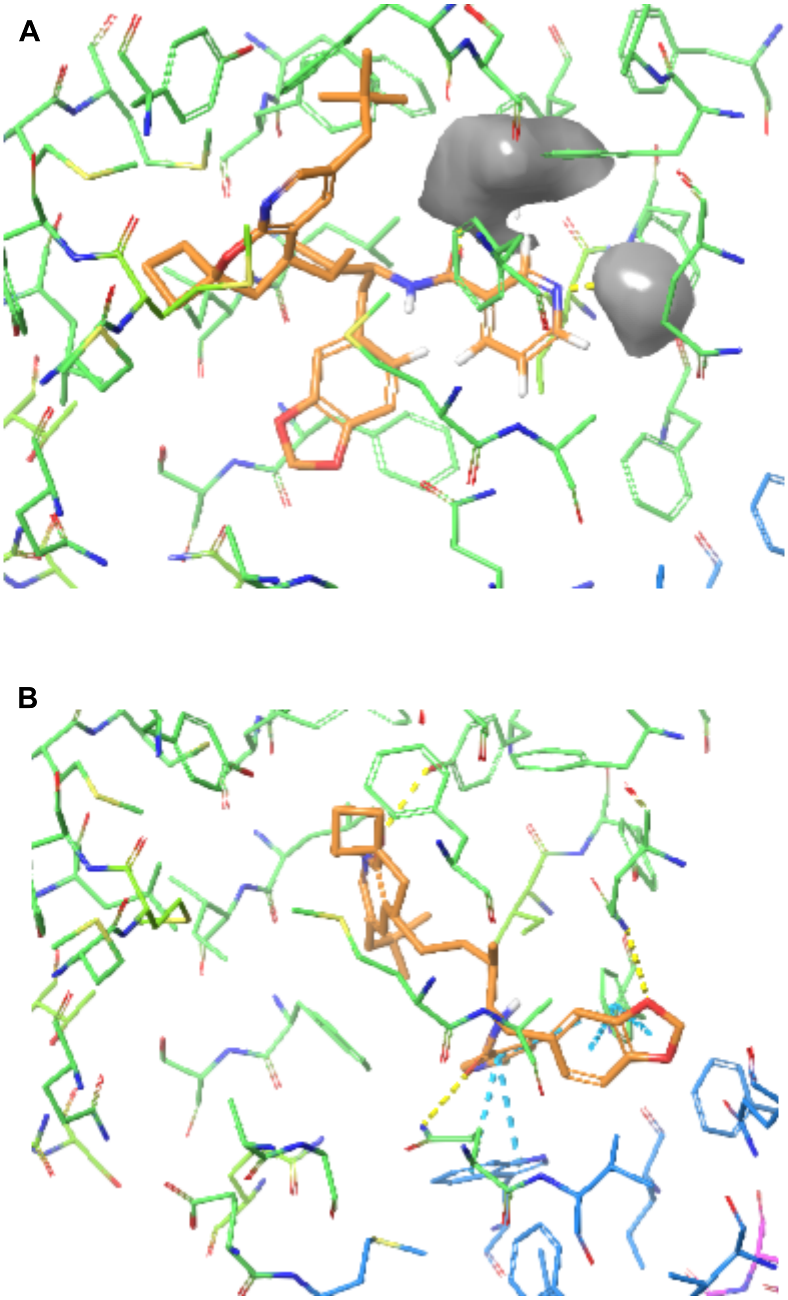

Figure S13. Compounds **9a** and **9b**: These molecules were synthesized as beta-secretase (BACE1) inhibitors that needed to be BBB penetrable. (A) The lowest energy pose for compound **9a** with a 3-pyridine group is shown together with surfaces for the Y307, which displays a H-bond with the pyridine N, and Y310, which displays a H-bond with the adjacent C=O. Substitution by F for H at the 2 or 4 position of the pyridine would be sterically hindered, leading to a different pose for compounds **9b** and **9c**. (B) The lowest energy pose for compound **9b** displays a strong pi-pi interaction with W232 in the vestibule domain. Compound **9b** also undergoes potent efflux by P-gp, so the lowest energy pose for **9b** (-46155) is comparable to, if not lower than, **9a** (-46146).

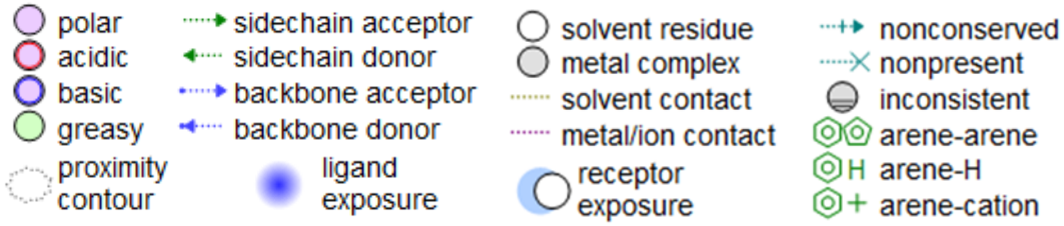

Figure S14. Legend for 2D Ligand Interaction Diagrams.

References

(1) Desai, P. V.; Raub, T. J.; Blanco, M. J. How hydrogen bonds impact P-glycoprotein transport and permeability. *Bioorg Med Chem Lett* **2012**, *22* (21), 6540-6548.
